# Supplementary material for: Capacitive piezotronics
Source: Nat Commun. 2026 Mar 26;17:4443. doi: 10.1038/s41467-026-71065-z (PMC13184101; doi:10.1038/s41467-026-71065-z)
Supplement: Supplementary file 1 — Supplementary Information [file 41467_2026_71065_MOESM1_ESM.pdf]

Supplementary Information for

Capacitive piezotronics

Luying Xu[1,2,3#], Zhuangzhuang Zhang[1,2,3#], Gaobo Wang[1,2,3], Yixuan He[1,2,3], Junyi Zhai[1,2,3], Weiguo Hu[1,2,3], Libo Chen[4], Longfei Wang[1,2,3]*, Shuhai Liu[5]*, and Zhong Lin Wang[1,2]*

[1] Beijing Institute of Nanoenergy and Nanosystems, Chinese Academy of Sciences, Beijing 101400, China.

[2] School of Nanoscience and Engineering, University of Chinese Academy of Sciences, Beijing 100049, China.

[3] Beijing Huairou Laboratory, Beijing, 101400, China.

[4] Division of Solid-State Electronics, Department of Electrical Engineering, Uppsala University, Uppsala 75121, Sweden.

[5] Institute of Nanoscience and Nanotechnology, School of Materials and Energy, Lanzhou University, 730000 Lanzhou, Gansu, China.

[#]These authors contribute equally to this work.

*Corresponding author. E-mail: lfwang12@binn.cas.cn, liushuhai1991@live.cn, zhong.wang@mse.gatech.edu.

This PDF file includes

Supplementary Notes 1-7

Supplementary Figures 1-30

References

Supplementary Note 1 | Comparison of sensitivity of capacitive piezotronic devices and commercial capacitive pressure sensors

To assess the performance on pressure sensing of our capacitive piezotronic devices, we compared it to two commercial MEMS capacitive pressure sensors: SCB10H-B250 pressure sensor elements from Murata Manufacturing[1] and Protron Mikrotechnik capacitive pressure sensors[2]. The pressure range of the SCB10H-B250 is 100-2500 kPa, exhibiting sensitivities of 0.13 fF/mbar at 100 kPa and 0.65 fF/mbar at 2500 kPa. And for the Protron Mikrotechnik sensor, the pressure ranges are 80-130 kPa and 200-700 kPa with sensitivities about 1×10^{-3}-4×10^{-3} fF/mbar and 0.5×10^{-3}-1.5×10^{-3} fF/mbar, respectively. Both of the two commercial sensors are similar in the pressure range to our devices, while our devices exhibit a much higher sensitivity: 20-110 fF/mbar (0-730 kPa) in a single Schottky junction, and 0.4-9.0 fF/mbar (0-2.42 MPa) in dual Schottky junctions (Supplementary Fig. 21). These results demonstrate the high sensitivity of capacitive piezotronic devices.

Supplementary Note 2 | Equivalent capacitance of single and dual Schottky junctions

First of all, we would like to clarify that whether in the devices with a single Schottky junction or dual Schottky junctions, the junction capacitance itself is theoretically independent on frequency. However, since these heterostructures cannot be regarded as ideal capacitors, their equivalent capacitances measured by an electrical instrument are not simply equal to their junction capacitances, but show complex dependence on frequency, junction resistance, junction capacitance, series resistance, etc.. Therefore, the relation between junction capacitance of heterostructure and its measured equivalent capacitance must be deeply investigated to strengthen the theoretical foundation of capacitive piezotronic effect proposed in our work, which aims at regulating junction capacitance via piezoelectric polarization.

2.1 Equivalent capacitance of a single Schottky junction

Here, we would like to clarify that the measured equivalent capacitance of a single Schottky junction exactly equals to its junction capacitance by applying appropriate test conditions. This fact allows for direct investigation of capacitive piezotronic effect in a single Schottky junction, greatly assisting us in intuitively understanding this effect. The detailed explanation is as follows:

As shown in Supplementary Fig. 14a, a single Schottky junction is described as a combination of junction resistance (R_j), junction capacitance (C_j) and series resistance (R_s). The corresponding impedance (Z_{single}) is:

$$Z_{single} = \frac{R_j}{1 + i\omega C_j R_j} + R_S, \qquad \text{(S-1)}$$

where ω is the angular frequency of alternating-current (AC) signal, and i is the imaginary unit. In our experiments, the frequency of AC signal ranges from 1 kHz to 10 kHz, and the single junction capacitance ranges approximately from 0.3 nF to 5 nF. Note that the junction resistance exhibits a strong dependence on the applied bias: generally, in the ~MΩ order under reverse bias while in the ~Ω order under forward bias [3-5]. The influence of junction resistance cannot be neglected since $(R_j)_{max} \approx 1/\omega C_j$. However, the influence of series resistance (generally in the ~Ω order[3-5]) can be overlooked under reverse bias because it is much smaller than the junction resistance (Supplementary Fig. 15). Thus, the single Schottky junction under reverse bias can be regarded as a single parallel connection of a resistor R_j and a capacitor C_j. The simplified impedance (Z_{re}) is:

$$Z_{re} = \frac{R_j}{1 + i\omega C_j R_j}. \qquad \text{(S-2)}$$

Under forward bias, the influence of series resistance must be taken into account, and the equivalent circuit of a single Schottky junction at this bias still maintains a complex structure. The corresponding

impedance (Z_{for}) is:

$$Z_{for} = \frac{R_j}{1 + i\omega C_j R_j} + R_S. \quad \text{(S-3)}$$

Note that there are two modes for investigating the impedance of the device in AC circuits: equivalent series circuit model (C_S) and equivalent parallel circuit model (C_P). The former one regards the entire circuit as a series connection of a capacitor ($C_{series\text{-}re}$ or $C_{series\text{-}for}$) and a resistor ($R_{series\text{-}re}$ or $R_{series\text{-}for}$):

$$Z_{re} = R_{series\text{-}re} + \frac{1}{i\omega C_{series\text{-}re}} \quad \text{(S-4)}$$

$$\text{or} \quad Z_{for} = R_{series\text{-}for} + \frac{1}{i\omega C_{series\text{-}for}}. \quad \text{(S-5)}$$

According to formulas (S-2) to (S-5), the equivalent capacitance of a single Schottky junction under reverse or forward bias measured by equivalent series circuit model is:

$$C_{series\text{-}re} = C_{series\text{-}for} = \frac{1 + \omega^2 C_j^2 R_j^2}{\omega^2 C_j R_j^2}. \quad \text{(S-6)}$$

And, the equivalent parallel circuit model regards the entire circuit as a parallel connection of a capacitor ($C_{parallel\text{-}re}$ or $C_{parallel\text{-}for}$) and a resistor ($R_{parallel\text{-}re}$ or $R_{parallel\text{-}for}$):

$$\frac{1}{Z_{re}} = i\omega C_{parallel\text{-}re} + \frac{1}{R_{parallel\text{-}re}} \quad \text{(S-7)}$$

$$\text{or} \quad \frac{1}{Z_{for}} = i\omega C_{parallel\text{-}for} + \frac{1}{R_{parallel\text{-}for}}. \quad \text{(S-8)}$$

According to formulas (S-2), (S-3), (S-7) and (S-8), the equivalent capacitance of a single Schottky junction under reverse or forward bias measured by equivalent parallel circuit model is:

$$C_{parallel\text{-}re} = C_j, \quad \text{(S-9)}$$

$$C_{parallel\text{-}for} = \frac{C_j R_j^2}{(R_j + R_S)^2 + \omega^2 C_j^2 R_j^2 R_S^2}. \quad \text{(S-10)}$$

Obviously, these two methods, while both capable of measuring the equivalent capacitance of a single Schottky junction, exhibit differences in their underlying principles and applications. In particular, the measured capacitance under reverse bias via equivalent parallel circuit model is exactly the single Schottky junction capacitance itself (S-9), showing independence on frequency, junction resistance and series resistance. Results measured under such conditions are quite suitable for studying the influence of capacitive piezotronic effect on interface width and junction capacitance. Therefore, discussion and analysis in our Fig. 3 are all based on the data measured under reverse bias via equivalent parallel circuit model.

2.2 Equivalent capacitance of dual Schottky junctions

The above derivation can also be extended to the case of dual Schottky junctions. Unfortunately, whatever the test conditions applied, the equivalent capacitance of dual Schottky junctions showing complex dependence on frequency. Therefore, it is necessary to investigate the *C-V* characteristics of dual Schottky junctions under different frequencies. The detailed explanation is as follows:

For dual Schottky junctions, which is composed of two single Schottky junctions (L and R) connected in series, the equivalent circuit is shown in Supplementary Fig. 14b. Here, additional series resistance in the circuit is also overlooked ($R_S = 0\ \Omega$). Since whether under forward or reverse bias, dual Schottky junctions always exhibits a much higher junction resistance than series resistance. On this basis, the impedance of dual Schottky junctions (Z_{dual}) is:

$$Z_{\text{dual}} = \frac{R_1}{1 + i\omega C_1 R_1} + \frac{R_2}{1 + i\omega C_2 R_2}, \tag{S-11}$$

where R_1 and R_2 are the junction resistances of the Schottky junctions L and R, C_1 and C_2 are the junction capacitances of the Schottky junctions L and R.

In the case of equivalent series circuit model, the entire circuit is regarded as a series connection of a capacitor ($C_{\text{series-dual}}$) and a resistor ($R_{\text{series-dual}}$):

$$Z_{\text{dual}} = R_{\text{series-dual}} + \frac{1}{i\omega C_{\text{series-dual}}}. \tag{S-12}$$

According to the formula (S-11) and (S-12), the equivalent capacitance of dual Schottky junctions measured by equivalent series circuit model is:

$$C_{\text{series-dual}} = \frac{\left(1 + \omega^2 C_1^2 R_1^2\right)\left(1 + \omega^2 C_2^2 R_2^2\right)}{\omega^2 C_1 R_1^2\left(1 + \omega^2 C_2^2 R_2^2\right) + \omega^2 C_2 R_2^2\left(1 + \omega^2 C_1^2 R_1^2\right)}. \tag{S-13}$$

And, in the case of equivalent parallel circuit model, the entire circuit is regarded as a parallel connection of a capacitor ($C_{\text{parallel-dual}}$) and a resistor ($R_{\text{parallel-dual}}$):

$$\frac{1}{Z_{\text{dual}}} = i\omega C_{\text{parallel-dual}} + \frac{1}{R_{\text{parallel-dual}}}. \tag{S-14}$$

According to the formula (S-11) and (S-14), the equivalent capacitance of dual Schottky junctions measured by equivalent parallel model is[6,7]:

$$C_{\text{parallel-dual}} = \frac{\omega^2 R_2^2 C_1 C_2 (C_1 + C_2) + C_1 + C_2 r^2}{\omega^2 R_2^2 (C_1 + C_2)^2 + (1 + r)^2}, \tag{S-15}$$

where r is the ratio of the junction resistances R_2 and R_1.

Obviously, whether equivalent series circuit model or equivalent parallel circuit model is used, the equivalent capacitance of dual Schottky junctions always shows dependence on the frequency of AC signal. Therefore, the investigation on the dispersive *C-V* characteristics of dual Schottky junctions is necessary in our work. Note that the experimental results in Fig. 4 are measured by equivalent parallel circuit model. Subsequent analyses of the dispersive *C-V* characteristics are all based on this model. According to formula (S-15), we can get the equivalent capacitance of dual Schottky junctions at low-frequency limit ($C_{\omega\text{-low}}$, ω tends to zero) and its equivalent capacitance at high-frequency limit ($C_{\omega\text{-high}}$, ω tends to infinity):

$$C_{\omega\text{-low}} = \frac{C_1 + r^2 C_2}{(1+r)^2}, \tag{S-16}$$

$$C_{\omega\text{-high}} = \frac{C_1 C_2}{C_1 + C_2}. \tag{S-17}$$

The physical quantities mentioned above can be respectively described by

$$R_1 = \left(\frac{kT}{qI_{S1}}\right)\exp(-qV_1/kT), \tag{S-18}$$

$$R_2 = \left(\frac{kT}{qI_{S2}}\right)\exp(qV_2/kT), \tag{S-19}$$

$$C_1 = \left[\frac{q\varepsilon_1 N_1}{2(V_{bi1} - V_1)}\right]^{\frac{1}{2}}, \tag{S-20}$$

$$C_2 = \left[\frac{q\varepsilon_2 N_2}{2(V_{bi2} + V_2)}\right]^{\frac{1}{2}}, \tag{S-21}$$

$$r = \frac{R_2}{R_1} = \left(\frac{I_{S1}}{I_{S2}}\right)\exp\left(\frac{qV}{kT}\right), \tag{S-22}$$

$$\frac{I_{S1}}{I_{S2}} = \exp\frac{\varphi_2 - \varphi_1}{kT}, \tag{S-23}$$

$$V_1 = \frac{V}{1+r}, \tag{S-24}$$

$$V_2 = \frac{rV}{1+r}, \tag{S-25}$$

where k is Boltzmann constant, T is the temperature, q is the elementary charge, I_{S1} and I_{S2} are the saturation currents of the Schottky junctions L and R, V_1 and V_2 are the voltage drops across the Schottky junctions L and R, V is the applied direct-current (DC) bias, ε_1 and ε_2 are the dielectric constants of the Schottky junctions L and R, N_1 and N_2 are the carrier concentrations of the Schottky junctions L and R, V_{bi1} and V_{bi2} are the built-in electric potentials of the Schottky junctions L and R, φ_1 and φ_2 are the interface barrier heights of the Schottky junctions L and R. According to formula

(S-16) and (S-17), we plot the *C*-*V* characteristics of dual Schottky junctions under different AC settings (Supplementary Fig. 14c). The low-frequency *C*-*V* characteristics exhibit two peaks, while present only one peak at high-frequency. The similarity between theoretical calculations and experimental results demonstrates the validity of the theoretical model mentioned above (Fig. 4b).

Supplementary Note 3 | Capacitive piezotronic modification on *C-V* characteristics of a single Schottky junction

3.1 Built-in electric potential of a single Schottky junction

In a single Schottky junction, the difference in Fermi electric potential of metal and semiconductor is referred as built-in potential and is denoted by V_{bi}. The built-in potential is one of the key parameters to describe the band energy structure in Schottky junction and the interface barrier height (φ) and width (W) are closely related to it[8],

$$\varphi = qV_{bi} + \varphi_n, \tag{S-26}$$

$$W = \sqrt{\frac{2\varepsilon_S(V_{bi} + V_R)}{qN_d}}, \tag{S-27}$$

where φ_n is the difference in Fermi energy level and conduction band energy level of the semiconductor, ε_S is the dielectric constant of the semiconductor, V_R is the reverse bias applied to the Schottky junction and N_d is the carrier concentration. According to the relationship between junction capacitance (C_j) and interface width mentioned in main text, we can derive the relationship:

$$\left(\frac{1}{C_j/S}\right)^2 = \left(\frac{W}{\varepsilon_S}\right)^2 = \frac{2(V_{bi} + V_R)}{q\varepsilon_S N_d}, \tag{S-28}$$

where S is the area of Schottky interface. Generally, the $1/C^2$-V characteristics are measured to get the built-in potential (intercept of V-axis) and the carrier concentration (slope) of the Schottky junction.

Considering the existence of piezoelectric polarization, the negative or positive polarization charges at interfaces will increase or decrease the built-in potential, respectively (Supplementary Fig. 16). To verify this idea, we measured the $1/C^2$-V characteristics of a single Schottky junction (Supplementary Fig. 16c) and performed a curve fitting using formula (S-28) (Supplementary Fig. 17). Results show that the dielectric constant of the semiconductor is almost no changed and the carrier concentration in it is ~8×10^{16} cm^{-3}. And, the built-in potential rises from 0.53 V to 0.59 V as negative polarization charges increases, which is consistent with our prediction.

3.2 Performance of capacitive piezotronic devices under different bias

We firstly analyzed the *C-V* characteristics of a single Schottky junction with built-in potential V_{bi0} in Supplementary Fig. 18. As the reverse bias increases, the depletion region of the junction tends to be

larger and thus the interface width increases, leading to a reduction in junction capacitance. When the reverse bias is sufficiently high to make the junction to be completely depleted, the junction capacitance tends to be a constant since the depletion region will not change anymore. Thus, the slope of C-V curve gradually decreases when the reverse bias increases. Similarly, as the forward bias increases, the interface width becomes narrower and thus the junction capacitance increases. When the bias gets close to the flat band voltage ($V_{\mathrm{flat}} = V_{\mathrm{bi0}}$), the capacitance tends to infinity rapidly. Accordingly, the slope of C-V curve increases rapidly as the forward bias increases.

From the perspective of capacitive piezotronics, when a loading force is applied to the Schottky junction along the polarization c-axis of the piezoelectric semiconductor, the negative polarization charges will be generated at the interfaces. As a result, the built-in potential will increase from V_{bi0} to V_{bi}', leading to the increment in interface width and reduction in junction capacitance. And, the C-V characteristics of the single Schottky junction will exhibit a positive offset (Supplementary Fig. 18):

$$\left(\frac{1}{C/S}\right)^2 = \left(\frac{W}{\varepsilon_{\mathrm{S}}}\right)^2 = \frac{2(V_{\mathrm{bi0}} + \Delta V + V_{\mathrm{R}})}{q\varepsilon_{\mathrm{S}} N_{\mathrm{d}}} = \frac{2(V_{\mathrm{bi}}' + V_{\mathrm{R}})}{q\varepsilon_{\mathrm{S}} N_{\mathrm{d}}}, \tag{S-29}$$

where ΔV is the change of built-in potential induced by piezoelectric polarization charges. Note that the change in capacitance is significantly different under different bias, despite of the same external loading force. It is due to the nonlinear dependence between the junction capacitance and the bias, when the depletion layer tends to be completely depleted or the bias gets close to V_{flat}. Thus, it can be concluded that, under the same loading force, the change in capacitance mainly depends on the slope of C-V curve. As mentioned above, the slope increases as the forward bias increases while decreases as the reverse bias increases. Since the capacitive sensor based on the Schottky junction generally operates at reverse bias, the best operating area of capacitive piezotronic device is the low reverse bias region.

Supplementary Note 4 | Capacitive piezotronic modification on low-frequency *C-V* characteristics of dual Schottky junctions

4.1 Physical mechanism underlying low-frequency *C-V* characteristics of dual Schottky junctions

According to formula (S-16), the low-frequency *C-V* characteristics exhibit an evident dependence on the ratio of junction resistances (r). To further clarify this, we have neglected the influence of junction capacitance by making a simplification that C_1 = C_2 = 1 F (independent on bias), and provided the simplified *C-V* curve and the corresponding original *C-V* curve in Supplementary Fig. 19a. Results show that the simplified curve exhibits a valley point, which locates exactly at the position where the valley point of the original *C-V* curve locates. And, it can be concluded that the valley point occurs only when $r = 1$, which means the two junction resistances are equal here. This conclusion also holds true whatever the junction is stressed. These findings prove that the valley point in low-frequency *C-V* characteristics of dual Schottky junctions results from the change of r, and only occurs when the two junction resistances are equal. The conclusions above can also be obtained by detailed mathematical derivations[6].

However, the peak in low-frequency curves cannot be simply attributed to the influence of r. Actually, it is dominated by the capacitance of reverse-biased junction. As shown in Supplementary Fig. 19b, we provided the capacitance variation of each junction when the whole device based on dual Schottky junctions is biased. For junction L, it is forward-biased when a positive voltage is applied on the device, possessing a much smaller junction resistance compared to the reverse-biased junction R. Thus, the voltage drop across junction L is nearly zero and its capacitance has almost no change. Conversely, the junction L is reverse-biased when a negative voltage is applied, possessing a much higher resistance than the forward-biased junction R. The voltage applied to the whole device almost entirely drops across the junction L and the junction capacitance decreases rapidly due to its increased interface width. Similar analysis is also applicable to junction R. By comparing the low-frequency *C-V* characteristics of dual Schottky junctions and the capacitance variation of each junction, it can be obtained that the peak occurs when the corresponding reverse-biased junction takes over the dominant role of r as the bias increases. Therefore, it can be concluded that the peaks in low-frequency *C-V* curves is mainly dominant by the reverse-biased junction.

4.2 Capacitive piezotronic modification on the valley point in low-frequency *C-V* curves and

calculation of the interface widths in dual Schottky junctions

Based on analysis above, the valley in low-frequency *C-V* curve occurs only at the bias where the resistances of the dual Schottky junctions are equal ($R_1 = R_2$), which also represents the interface barrier heights are equal here ($\varphi_1 = \varphi_2$). Generally, this symmetry condition $R_1 = R_2/\varphi_1 = \varphi_2$ can be satisfied at a specific bias (which may coincide with zero bias if the configuration of dual Schottky junctions is almost symmetric). When the force applied on one of the junctions, the induced negative polarization charges will increase its interface barrier height and width and the specific bias that satisfying the symmetry condition will change, with the valley shifts and a variation in *C-V* curve being induced (Supplementary Fig. 20a). These features provide a basis for calculating the interface barrier heights and widths in dual Schottky junctions[6,7].

In devices of dual Schottky junctions, we can assume that $\varepsilon_1 = \varepsilon_2 = \varepsilon$, $N_1 = N_2 = N_d$. The interface widths of junction L (W_1) and R (W_2) at zero bias satisfy:

$$W_1 = \sqrt{\frac{2\varepsilon_S V_{bi1}}{qN_d}}, \quad \text{(S-30)}$$

$$W_2 = \sqrt{\frac{2\varepsilon_S V_{bi2}}{qN_d}}. \quad \text{(S-31)}$$

Suppose the junction L is stressed and the junction R is unstressed, the piezoelectric polarization charges will increase the built-in potential of junction L and thus $V_{bi1} > V_{bi2}$, $\varphi_1 > \varphi_2$. Under this condition, the valley point, which occurs only when the interface barrier heights of the dual Schottky junctions are equal, will shift to the bias (V_{valley}):

$$V_{valley} = \frac{\varphi_1 - \varphi_2}{q} = V_{bi1} - V_{bi2}. \quad \text{(S-32)}$$

Under this bias condition, the voltage drops across junction L ($V_{1\text{-valley}}$) and R ($V_{2\text{-valley}}$) are equal according to formulas (S-22) to (S-25):

$$\frac{V_{2\text{-valley}}}{V_{1\text{-valley}}} = \frac{R_{2\text{-valley}}}{R_{1\text{-valley}}} = \exp\left(\frac{\varphi_2 - \varphi_1}{kT}\right)\exp\left(\frac{qV_{valley}}{kT}\right) = 1, \quad \text{(S-33)}$$

$$V_{1\text{-valley}} = V_{2\text{-valley}} = \frac{V_{valley}}{2} = \frac{V_{bi1} - V_{bi2}}{2}, \quad \text{(S-34)}$$

where $R_{1\text{-valley}}$ and $R_{2\text{-valley}}$ are the junction resistances of the Schottky junctions L and R at the valley point. In this way, the interface widths of junction L ($W_{1\text{-valley}}$) and R ($W_{2\text{-valley}}$) at valley point are:

$$W_{1\text{-valley}} = \sqrt{\frac{2\varepsilon_S\left(V_{bi1} - V_{valley}\right)}{qN_d}} = \sqrt{\frac{\varepsilon_S\left(V_{bi1} + V_{bi2}\right)}{qN_d}}, \quad \text{(S-35)}$$

$$W_{2\text{-valley}} = \sqrt{\frac{2\varepsilon_S\left(V_{bi2} + V_{valley}\right)}{qN_d}} = \sqrt{\frac{\varepsilon_S(V_{bi1} + V_{bi2})}{qN_d}}. \tag{S-36}$$

Here, we define $W_{1\text{-valley}} = W_{2\text{-valley}} = W_{valley}$. According formulas (S-30), (S-31), (S-35) and (S-36), we can obtain the relation:

$$\frac{W_1^2 + W_2^2}{2} = W_{valley}^2, \tag{S-37}$$

Similar derivation can be conducted under the case that the junction R is stressed. To verify these relations, simulations on *C-V* characteristics of dual Schottky junctions with symmetric/asymmetric configuration are performed in Supplementary Fig. 20b, c. The results show that V_{valley} is strictly equal to the difference in interface barrier heights and the capacitance at the valley point gradually decreases as one of the interface barrier heights increases, being consistent with formula (S-32) and (S-37). In this way, the change in interface barrier heights and widths induced by piezoelectric polarization potential can be quantitatively analyzed (Supplementary Fig. 21).

4.3 Capacitive piezotronic modification on the peaks in low-frequency *C-V* curves of dual Schottky junctions

It is obvious that the variation of valley points in low-frequency *C-V* curves measured in our experiments (Fig. 4c, f and Supplementary Fig. 21a-d) can be well explained by simulation results in Supplementary Fig. 20b, c. However, the peaks of the *C-V* curves exhibit an inconsistency between the experimental results and the theoretical simulations. Specifically, the capacitance at the peak shifting closer to zero bias shows an increment, rather than remain unchanged as simulated above. In the former simulations, we supposed that the change in built-in electric potential (V_{bi}) and interface width (W) perfectly follows a steady relationship according to formula (S-27), which is derived from the 1D Poisson's equation:

$$\frac{d^2\psi(x)}{dx^2} = \frac{1}{\varepsilon_S}[qN_d] \tag{S-38}$$

where $\psi(x)$ is the electric potential distribution along the x-axis, as shown in Supplementary Fig. 22a. Notably, the validation of formula (S-27) requires that the curvature of $\psi(x)$ does not change, so that the change in built-in electric potential and interface width could be perfectly matched under an external stress. To meet this requirement, we actually set a hypothesis that the piezoelectric charges distribute normally in a very thin layer and have little influence on the potential curvature. This

hypothesis is valid as we explain the single junction model, but not enough in the dual junction model.

The reality is that, as the force increases, the piezoelectric polarization charges change the distribution of free carriers and donors, simultaneously changes the effective charge concentration (N_{eff}) significantly:

$$\frac{\mathrm{d}^2\psi(x)}{\mathrm{d}x^2} = \frac{q}{\varepsilon_{\mathrm{S}}}[N_{\mathrm{d}} + \Delta N] = \frac{q}{\varepsilon_{\mathrm{S}}}N_{\mathrm{eff}} \qquad \text{(S-39)}$$

where ΔN is the variation in the concentration. In this way, the curvature of $\psi(x)$ increases and the band bending becomes much steeper. Consequently, the depletion region essentially narrows to accommodate the sharp potential drop, even though the total barrier is larger (Supplementary Fig. 22a). This narrowed depletion regions results in the mismatch between built-in electric potential and interface width, leading to a narrower interface width under same conditions. So, we have induced the mismatch between built-in electric potential and interface width into our simulations (Supplementary Fig. 22b). Results show that the peak shifting closer to zero bias shows an increment, and the other peak shows a reduction, which is consistent with our experiments.

Supplementary Note 5 | Capacitive piezotronic modification on the peaks in high-frequency *C-V* characteristics of dual Schottky junctions

Under high frequency settings, the influence of junction resistances is reduced according to the formula (S-17), and thus the valley in *C-V* curve induced by the ratio of the junction resistances disappears, leading to only one peak centering at near-zero bias region in the curve. Similar to the case under low-frequency settings, the peak locates at a specific bias (which may coincide with zero bias if the configuration of dual Schottky junctions is almost symmetric) under zero-strain condition, while exhibits a shift when the configuration of dual Schottky junctions is modified by the force-induced piezoelectric polarization potential. This is demonstrated by both experimental results and theoretical simulations (Fig. 4 and Supplementary Fig. 23a). However, the variation in peak value exhibits inconsistency between the experimental and theoretical results: the peak increases in the experiments while decreases in the simulations. This is due to the fact that the effective charge concentration (N_{eff}) of the stressed junction increases under loading force, as demonstrated in Supplementary Note 4.3. The N_{eff}-modified theoretical results have been provided in Supplementary Fig. 23b, which shows an increasing peak and is consistent with the experimental results.

Supplementary Note 6 | Strain-induced band modulation in GaN

6.1 *C-V* characteristics of GaN with interface traps

In semiconductor devices based on Schottky contact, MIS contact or *p-n* junction, etc., the presence of interface states/traps introduces localized levels in bandgap, which can capture and release the charged carriers. Under forward bias, the efficiency of charge capture and release by interface traps is enhanced due to the increased unoccupied interface traps, resulting in the generation of an excess capacitance (C_{it})[9-11]. To demonstrate this phenomenon, *C-V* measurements were conducted on dual Schottky junctions with interface traps under different AC settings (Supplementary Fig. 24). Note that the junction L is forward-biased under positive bias, while the junction R is forward-biased under negative bias. The excess capacitance is observed under both negative and positive bias. Besides, as the frequency increases, the excess capacitance response should be weakened. This is because the time constant of the carrier capture/release process is too large (~ several hundred ms) to keep up with the high-frequency signals (> 10 kHz) [12], which is also confirmed by our experiments.

6.2 Strain-induced band modulation in GaN with interface traps

As is well known, mechanical strain can modulate the band structure of GaN[13-15], then further controlling the carrier dynamics at interface traps. Specifically, compressive strain can significantly decrease the conduction band-edge energy but only slightly increase the valence band-edge energy, leading to a narrower bandgap, a lower Fermi level and interface barrier height. Moreover, a lower Fermi-level introduces more unoccupied interface traps at Schottky interfaces, enhancing the charge capture/release efficiency and thus increasing the excess capacitance (Supplementary Fig. 25a). In contrast, tensile strain suppressing these processes and leading to a decrease in excess capacitance. Studies on heavy-doped GaN with interface traps support these theories (Supplementary Fig. 25b-e). As mentioned above, the junction L is forward-biased at positive bias and thus the corresponding excess capacitance occurs at positive bias; while the interface traps located at junction R induces the excess capacitance at negative bias. Results show that the corresponding excess capacitance increases when the external loading force is applied to the junction L or R, which is consistent with our prediction. However, researches on GaN with low carrier concentration exhibit an opposite trend: when the force is applied to one of the junctions, the corresponding excess capacitance is reduced (Supplementary Fig.

26). This phenomenon is attributed to the piezoelectric polarization, which cannot be fully screened in piezoelectric materials with low carrier concentration. Here, repetitive characterizations were carried out to exclude the influence of charge injection, ionic migration and etc. (Supplementary Fig. 27). From another perspective, the investigation into strain-induced band modulation further provides a more comprehensive understanding of capacitive piezotronics.

Supplementary Note 7 | Discussion and clarification of different force sensing mechanisms

Force sensors, which are used to monitor the mechanical signal including stress, strain, acceleration, torque, etc., are vitally important in mechanosensation, human-machine interfacing, and robotics. The working mechanisms of commercial force sensors mainly include capacitive effect, piezoelectric effect and piezoresistive effect. In this work, the capacitive piezotronic effect can also achieve the function of force sensing, serving as an unconventional mechanism of force sensing. Here, we have provided a tree cross graph to show the position of this work among force sensing mechanisms (Supplementary Fig. 28), and made a discussion on the difference between our capacitive piezotronic devices and traditional commercial devices.

(1) Capacitive force sensors

The sensing element in traditional capacitive sensors is a variable parallel plate capacitor, which is usually constructed by a movable electrode and a fixed electrode. The movable electrode is displaced under external force and thus the space between the two electrodes is changed. Note that the capacitance of parallel plate capacitor (C_{plate}) is defined as:

$$C_{\text{plate}} = \varepsilon_{\text{plate}} \frac{S_{\text{plate}}}{W_{\text{plate}}}, \tag{S-40}$$

where $\varepsilon_{\text{plate}}$ is the dielectric constant of the dielectric material between the two electrodes, S_{plate} is the area of the electrode and W_{plate} is the width between the two electrodes. In this way, the capacitance of parallel plate capacitor can be regulated by changing the gap between electrodes via external force. This is the basic modulation mechanism underlying most capacitive sensors.

Obviously, this effect is different from the capacitive piezotronic effect proposed in our work. Traditional capacitive devices mainly depend on the change in the geometric structure of the devices without participation of interface polarization, while capacitive piezotronic effect is an interface effect that utilize piezoelectric polarization to modulate the interface width of heterostructure. Moreover, the materials applied in capacitive sensors mainly includes Si, ceramic, etc., while that applied in capacitive piezotronic devices must be non-centrosymmetric piezoelectric materials, such as ZnO, GaN, AlN, etc..

(2) Piezoelectric force sensors

The working mechanism of traditional piezoelectric sensors can be summarized as the generation of polarization charges and the harvest of induced charges. The sensing element in the traditional piezoelectric sensor is made of piezoelectric materials, such as SiO_2, $BaTiO_3$, $Pb(Zr,Ti)O_3$, etc..[16] An external force can induce a deformation in the sensing element, and thus result in the rearrangement and alignment of dipoles in the piezoelectric material. These bound charges further generate induced charges in external circuits and output an electric signal. Notably, there is another type of piezoelectric sensor that also uses capacitance as a key parameter. The polarization bound charges are produced on the surface of the sensing element, which could directly change the equivalent capacitance of the device.

Although this type of sensor seems like our capacitive piezotronics sensors, they are actually fundamentally different. Specifically, the force-induced polarization in a traditional piezoelectric sensor is utilized to induce bound charges on the surfaces of the sensing element, so as to control the equivalent capacitance of the dielectric layer (but not the junction). While in the capacitive piezotronic devices, the polarization aims at regulating the interface width of heterostructure, so as to change the junction capacitance. Therefore, traditional piezoelectric sensor is usually based on a metal-insulator-metal (MIM) structure, while capacitive piezotronic device mainly applies heterojunction structure, such MS junction, MIS junction and *p-n* junction.

(3) Piezoresistive force sensors

Strain applied to some specific materials, notably semiconductors, can modify their energy band structures and conductivities, allowing effective modulation on the resistance of the corresponding devices. This is the working mechanism of piezoresistive sensor. It is a volume effect without polarity and mainly focuses on the resistance of the devices. This is also obviously different from capacitive piezotronic effect.

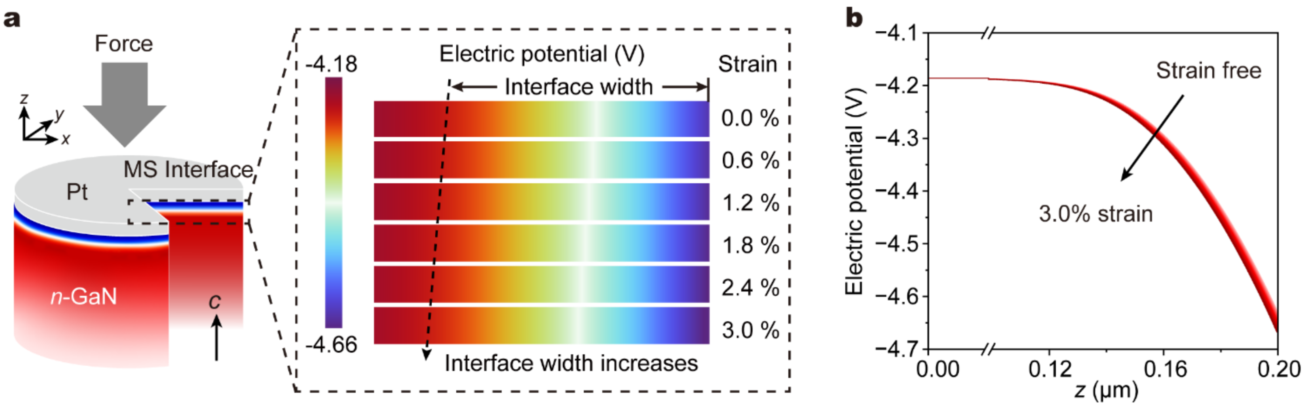

Supplementary Figure 1 | Simulations on electric potential at Metal-Semiconductor (MS) interface by FEM. a, Schematic diagram of Schottky interface and electric potential distribution at the interface under loading forces, showing the increment in the interface width as force increases. **b**, The calculated electric potential distributions along *z*-axis with strain ranging from 0% to 3%. The stepwise is 0.05%. The electric potential near the interface becomes higher as the loading force increases.

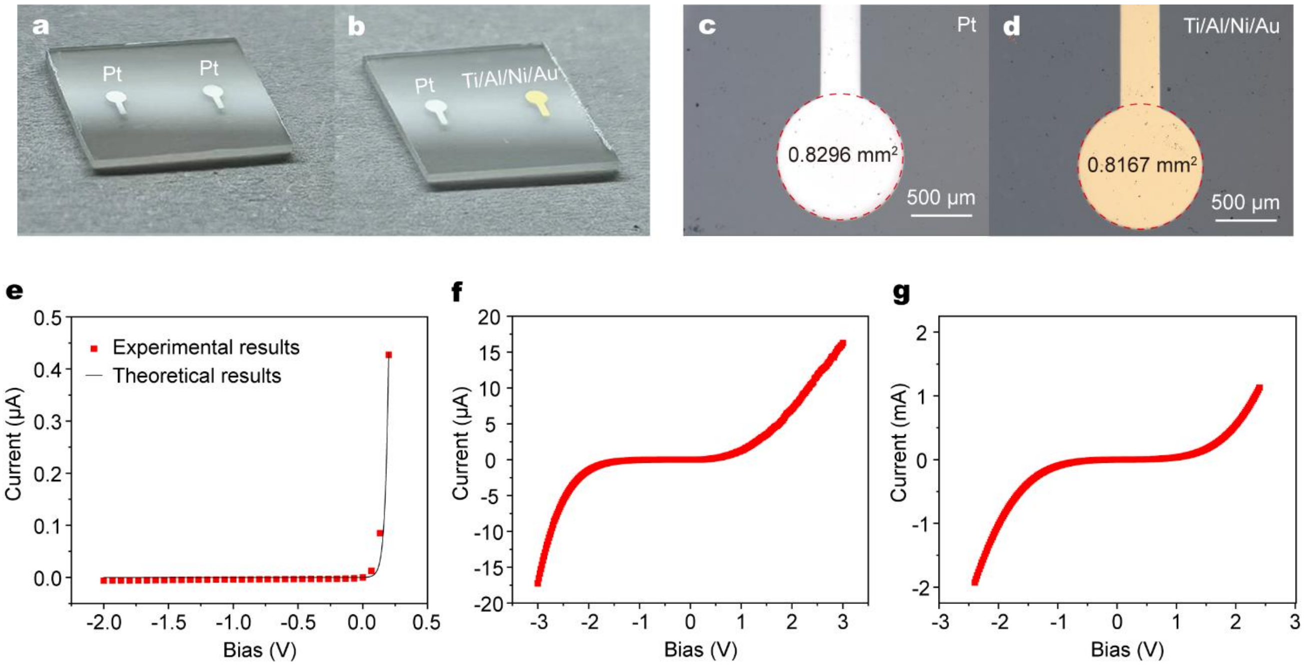

Supplementary Figure 2 | Characterization of capacitive piezotronic devices. a, The device based on dual Schottky junctions. **b**, The device based on single Schottky junction. **c**, The optical image of the Schottky contact (Pt/*n*-GaN). Force is applied on the circular area and the lead wires are connected to the strip area. The interface area S = 0.8296 mm^2. **d**, The optical image of the Ohmic contact (Ti/Al/Ni/Au/*n*-GaN). **e**, The *I*-*V* characteristic of single Schottky junction fabricated on low-carrier-concentration GaN. The experimental results fit well with the theoretical results in thermionic emission theory and the obtained interface barrier height is ~0.66 eV. **f,** The *I*-*V* characteristic of dual Schottky junctions fabricated on low-carrier-concentration GaN. **g,** The *I*-*V* characteristic of dual Schottky junctions fabricated on high-carrier-concentration GaN.

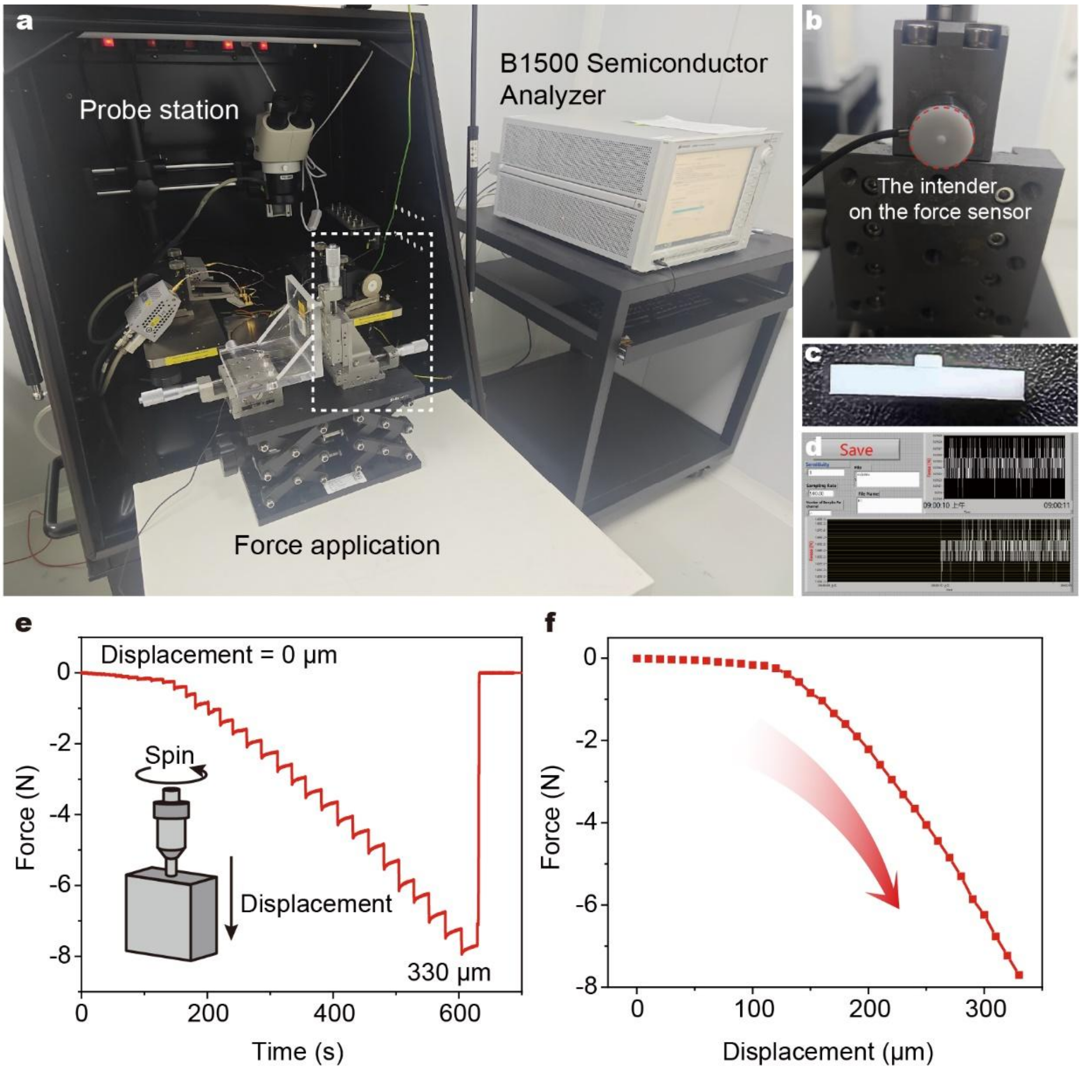

Supplementary Figure 3 | Measuring system. a, A total view of the measuring system. **b**, Force application and sensing module, containing an intender, a sensor and a translation stage. **c**, The intender. **d**, The software for data collection and processing. **e**, Real-time force monitoring as the displacement of translation stage increases. The step for each displacement is 10 μm. **f**, Loading force as a function of the displacement of translation stage. With this relationship, the applied force in experiments can be quantified by the displacement of translation stage.

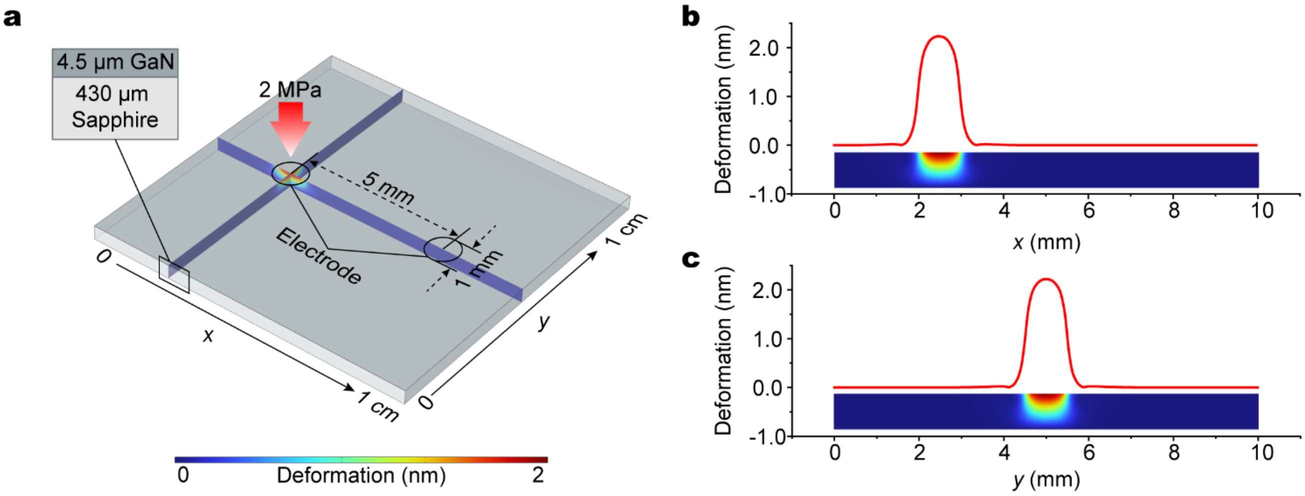

Supplementary Figure 4 | Analysis of the deformation of two-terminal devices under loading force. a, Deformation of the two-terminal device with 2 MPa pressure applied on one of the junctions. The deformed area is concentrated within a range of ~1 mm near the stressed junction. **b,** The deformation distribution along x-axis (y = 5 mm) at the top surface of GaN. Area near the unstressed junction (x ranges from 7 to 8 mm) has almost no change. **c,** The deformation distribution along y-axis (x = 2.5 mm) at the top surface of GaN.

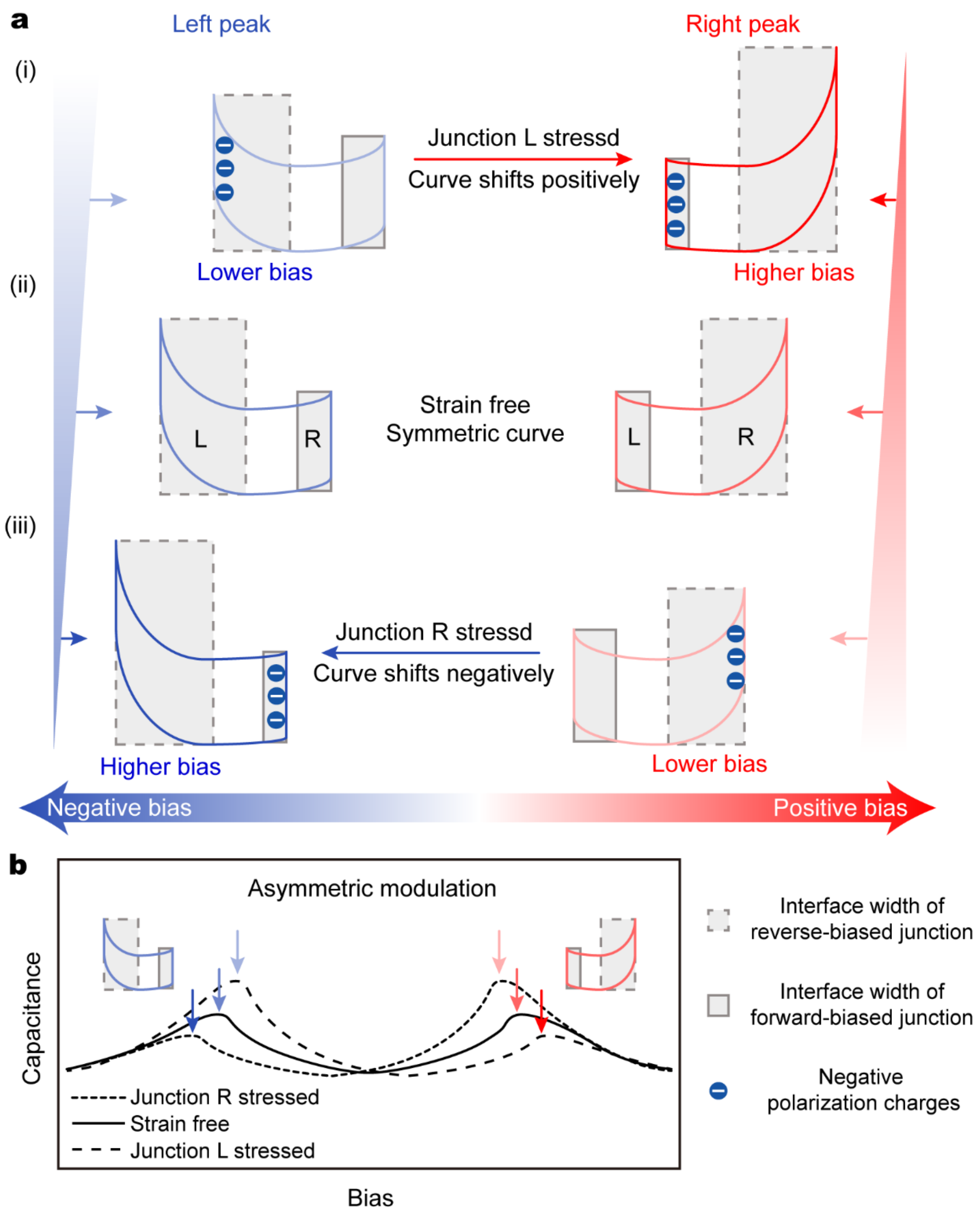

Supplementary Figure 5 | Interface width of dual Schottky junctions at peak position. a, Energy band structures corresponding to left (blue) and right (red) peaks in low-frequency curves. The junction L is reverse-biased at negative bias and forward-biased at positive bias, while the junction R is forward-biased at negative bias and reverse-biased at negative bias. When the force is applied to the junction L (i), the *C-V* curve shifts positively and the left peak occurs at lower negative bias and the right peak occurs at higher positive bias compared to the situation without force applied (ii). A lower negative bias reduces the interface width of reverse-biased junction (junction L) and a higher positive bias increases the interface width of reverse-biased junction (junction R). The change in energy band structure is opposite when force is applied to the junction R (iii). **b**, Asymmetric modulation on low-frequency *C-V* characteristics. The variation in peak capacitance is mainly dominated by the interface width of reverse-biased junction and is consistent with the mechanism mentioned in **a**.

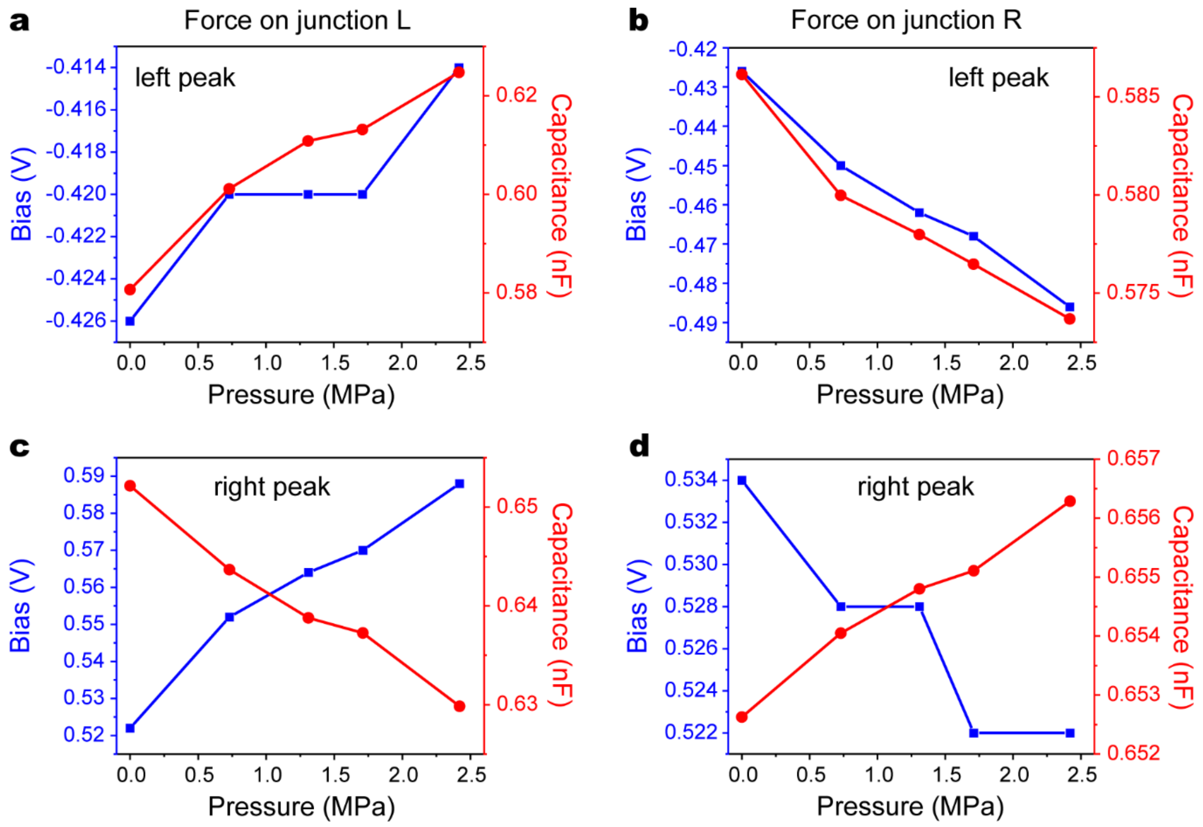

Supplementary Figure 6 | Variation in peaks in low-frequency *C-V* characteristics of dual Schottky junctions. a, **b**, The change of left peaks in Fig. 4c, f with force applied to junction L (**a**) and R (**b**). When the force is applied to the junction L, the left peak gradually shifts towards zero bias and its capacitance increases. When the junction R is stressed, the left peak gradually moves away from zero bias and its capacitance decreases. **c**, **d**, The change of right peaks in Fig. 4c, f with force applied to junction L (**c**) and R (**d**), contrary to the change of left peaks in **a**, **b**.

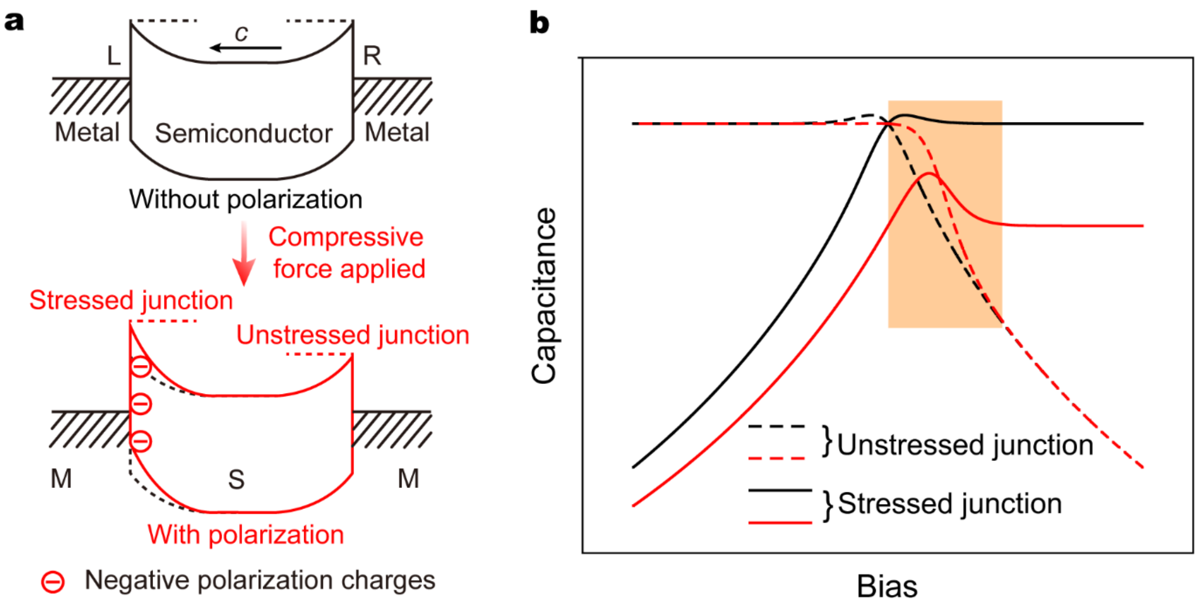

Supplementary Figure 7 | *C-V* characteristics of the stressed and unstressed Schottky junction. **a**, Schematics of junction L and R without force applied (black) and under loading force (red). **b**, *C-V* characteristics of the stressed junction L (solid lines) and the unstressed junction R (dashed lines) without force applied (black) and under loading force (red). The peaks in high-frequency curves occur in the bias region noted by the shaded area, where the capacitance of stressed junction decreases and that of unstressed junction increases.

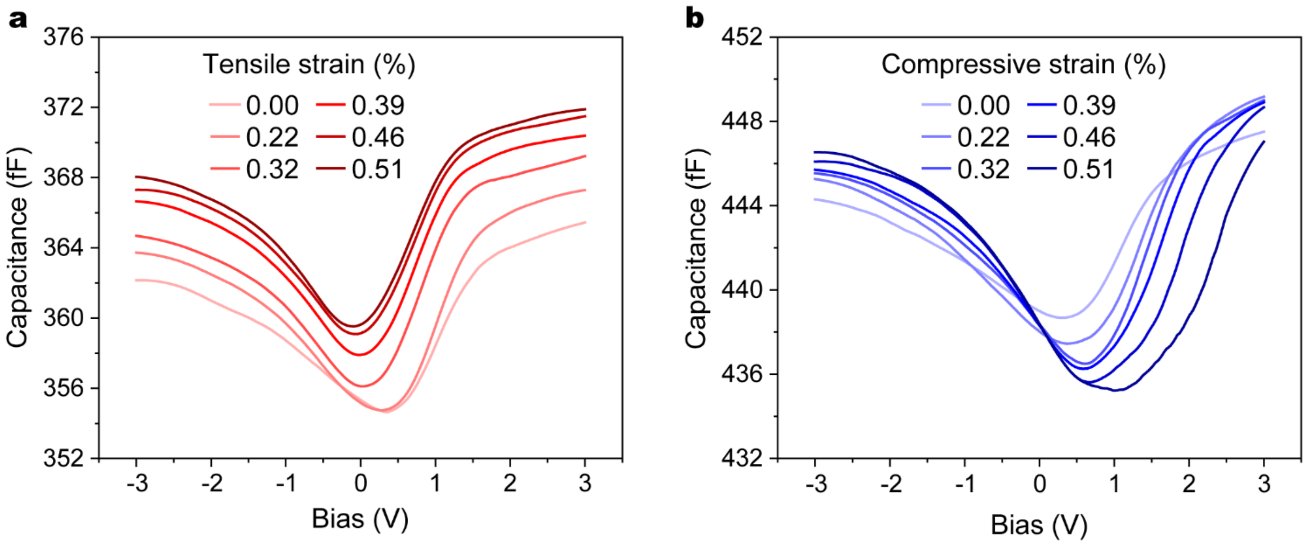

Supplementary Figure 8 | Capacitive piezotronics in ZnO. a, *C*-*V* characteristics measured in single crystal ZnO microwires under the increasing tensile strain. **b**, *C*-*V* characteristics measured in single crystal ZnO microwires under the increasing compressive strain. A completely overturned variation compared to **a** is observed.

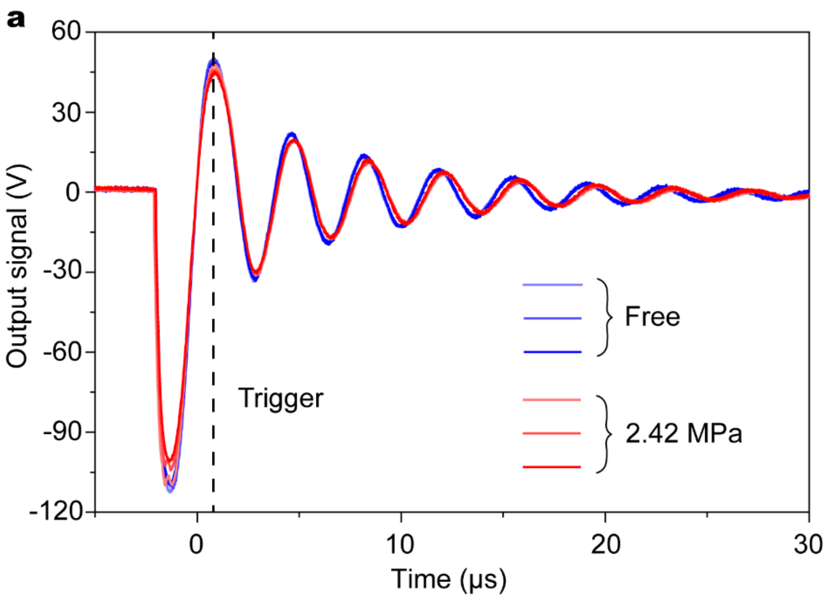

Supplementary Figure 9 | Repetitive measurements of the output signals of frequency modulation circuit. a, The oscillation signals in frequency modulation circuit in Figure 5b under repetitive measurements. **b**, Fast Fourier Transform spectrum of the oscillation signal shown in **a**. The change in oscillation frequency is ~9 kHz. Reproducibility is ensured by the repetitive measurements.

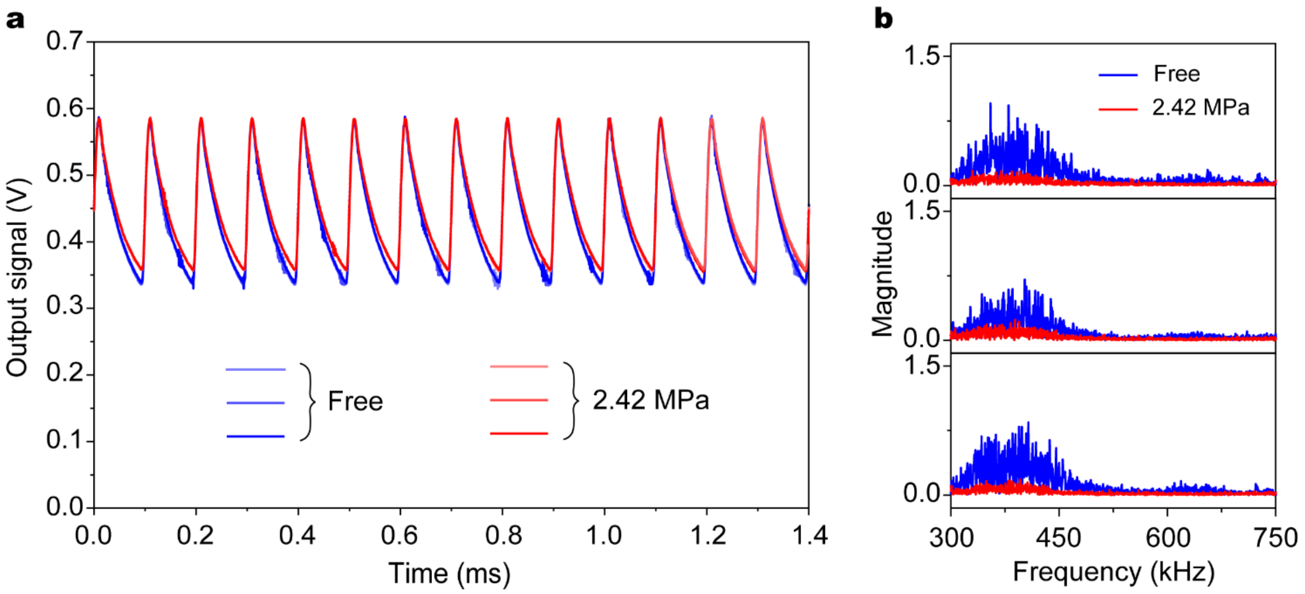

Supplementary Figure 10 | Repetitive measurements of the output signals of filtering circuit. a, The filtered signals in filtering modulation circuit in Fig. 5e under repetitive measurements. **b**, Fast Fourier Transform spectrum of the filtered signal shown in **a**. Reproducibility is ensured by the repetitive measurements.

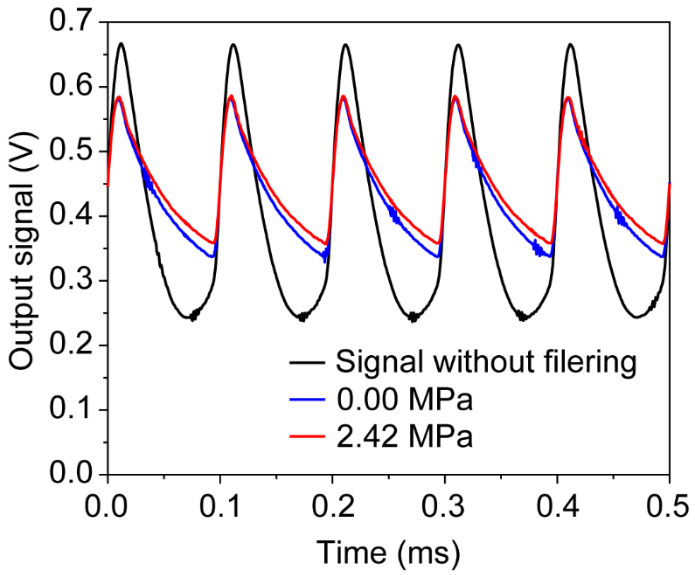

Supplementary Figure 11 | The filtering performance of the capacitive piezotronic device under different loading forces. The half-wave rectification on a 10 kHz sinusoidal wave signal results in the signal simultaneously possessing direct-current (DC) and alternating-current (AC) components (black). This signal can be partly filtered by the capacitive piezotronic device without force applied, however the high-frequency noise still exists (blue). Under a loading force, the filtering performance is enhanced and the high-frequency noise disappeared (red).

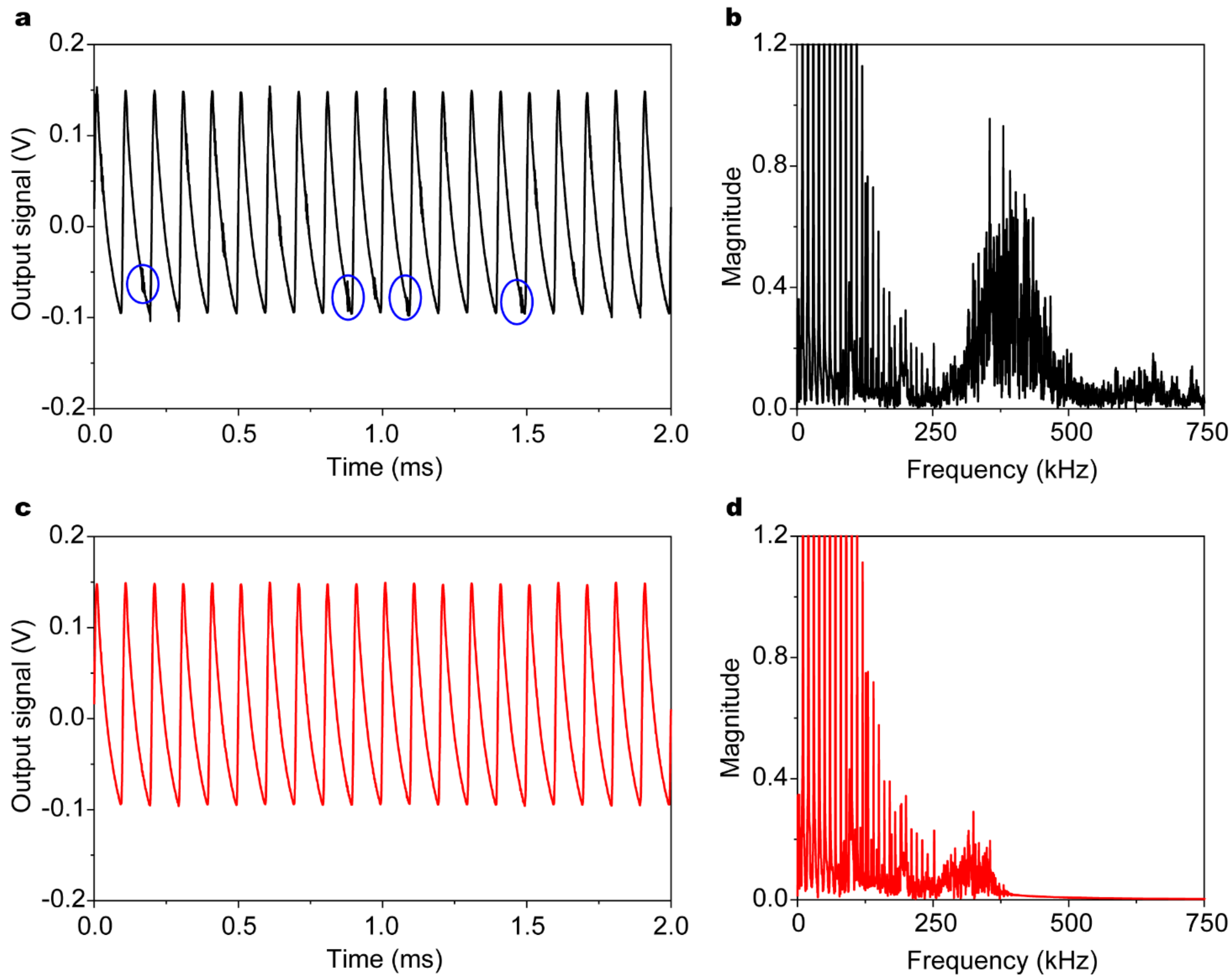

Supplementary Figure 12 | Analysis on high-frequency noise via filter function. a, The signal with high-frequency noise in filtering circuit. The high-frequency noise is noted by blue circles. **b**, Fast Fourier Transform spectrum of the signal in **a**. An obvious peak is located at ~300 kHz. **c**, The signal in **a** after applying a low-pass filter function. The high-frequency noise disappears and the signal becomes smoother. The cutoff frequency of the low-pass filter function is 250 kHz. **d**, Fast Fourier Transform spectrum of the signal in **c**. The peak located at ~300 kHz disappears. Results show that the high-frequency noise in filtering circuit indeed corresponds to the peak located at ~300 kHz in Fast Fourier Transform spectrum.

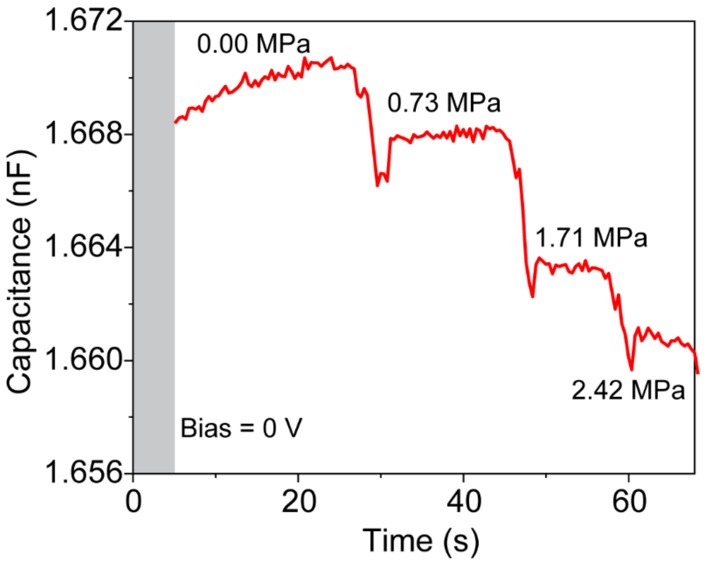

Supplementary Figure 13 | *C-t* characteristics of capacitive piezotronic devices. As the force increases, the negative polarization charges reduce the Schottky junction capacitance and the *C-t* characteristics exhibit a stepwise decrease, which is contrary to the variation in resistance under the same piezoelectric polarization.

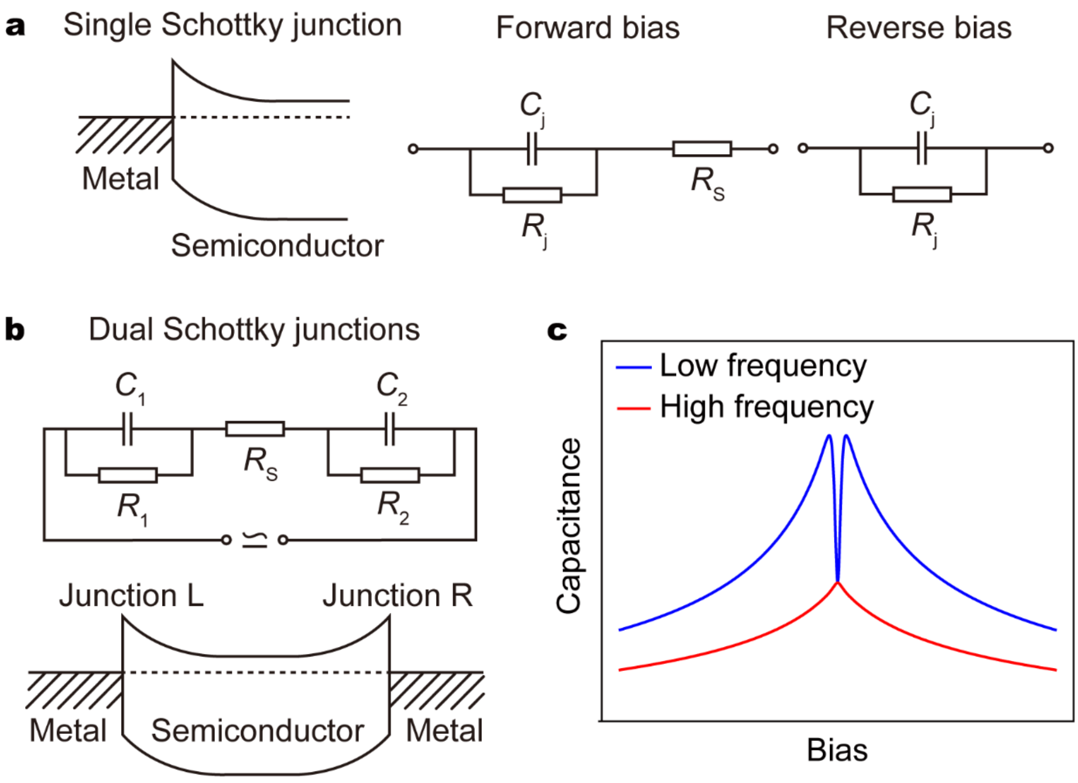

Supplementary Figure 14 | Theoretical model of single and dual Schottky junctions. a, The energy band structure of single Schottky junction and the corresponding equivalent circuit under forward and reverse bias. **b**, The equivalent circuit and energy band structure of dual Schottky junctions. Here, the influence of series resistance can be neglected ($R_S = 0\ \Omega$). **c**, Simulated C-V characteristics of dual Schottky junctions under low and high frequency settings. The two peaks in low-frequency C-V curve decrease until they degenerate into one peak as the frequency increases. C_j, R_j, R_S, C_1, C_2, R_1 and R_2 are all defined in Supplementary Note 2.

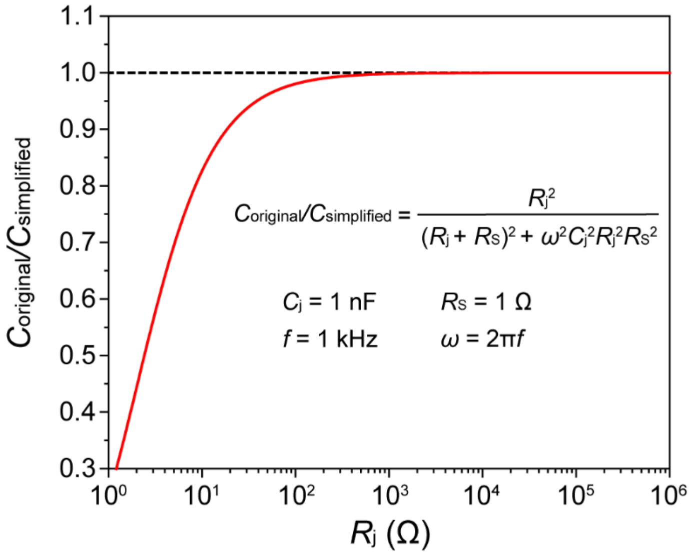

Supplementary Figure 15 | Comparation on equivalent capacitances of single Schottky junction with ($C_{original}$) and without ($C_{simplified}$) the influence of series resistance. The two equivalent capacitances tend to be consistent when the junction resistance is much larger than series resistance. So, the series resistance can be neglected under this condition. The parameters used in simulations are basically consistent with those applied in our experiments. C_j, R_j, R_S, ω and f are all defined in Supplementary Note 2.

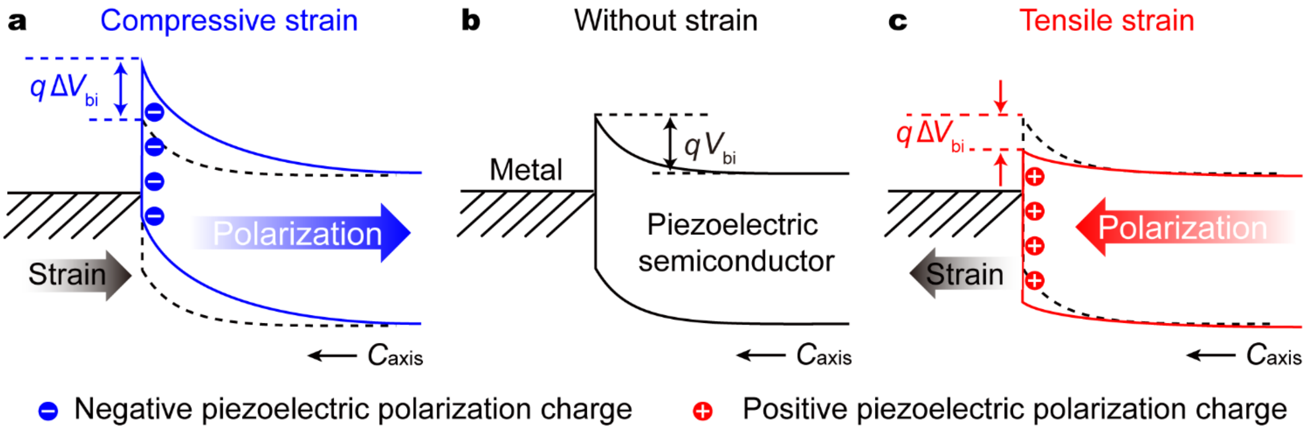

Supplementary Figure 16 | Modulation mechanism of piezoelectric polarization on the built-in potential of Schottky junction at compressive strain (a), strain free (b) and tensile strain (c) conditions. Here, q is the elementary charge, V_{bi} refers to the built-in electric potential and ΔV_{bi} refers to the change in the built-in electric potential.

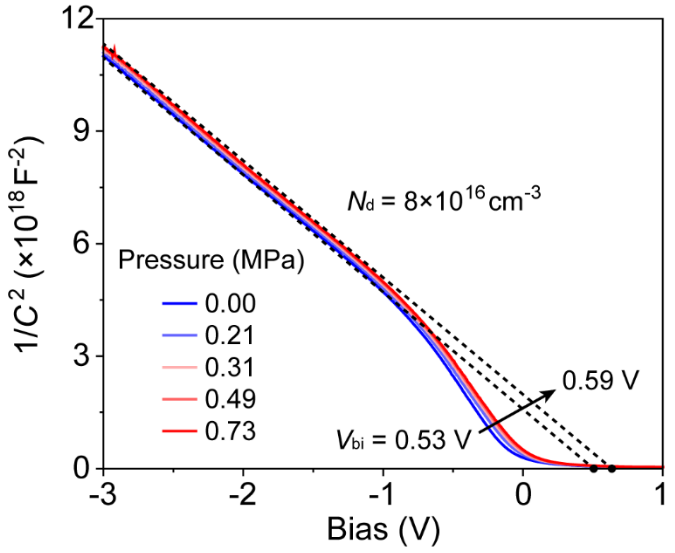

Supplementary Figure 17 | Built-in potential of Schottky junction under different loading forces. The $1/C^2$-V curves of a single Schottky junction and the corresponding linear fitting curves are shown under different loading forces. The built-in potential and carrier concentration are calculated by the formula in the figure. Here, V_{bi} refers to the built-in electric potential and N_d refers to the carrier concentration.

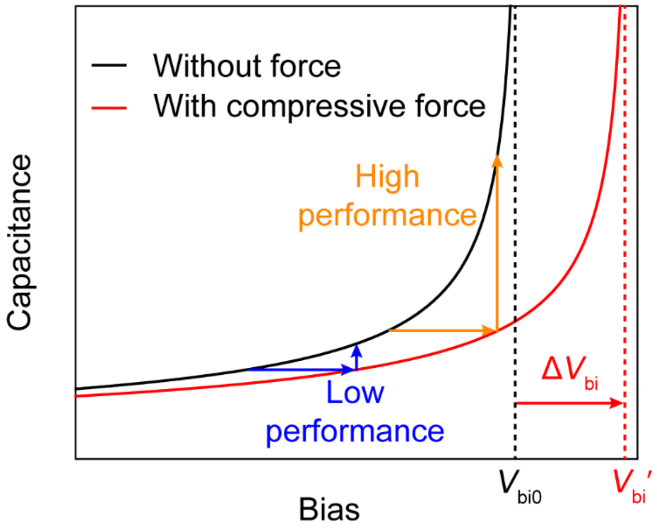

Supplementary Figure 18 | Performance of capacitive piezotronic effect under different bias. The change in built-in potential induced by piezoelectric polarization (ΔV_{bi}) results in an offset of *C*-*V* curve. V_{bi0} and V_{bi}' represent the built-in potential without stain and with compressive strain, respectively. Capacitive piezotronic effect exhibits higher modulation performance as the reverse bias decreases and the forward bias increases.

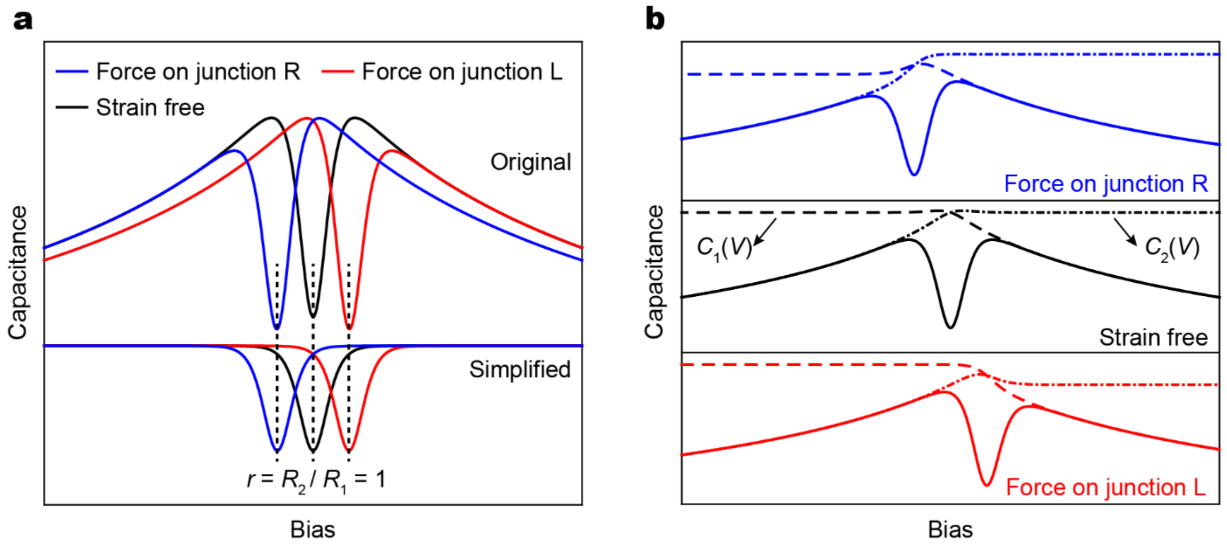

Supplementary Figure 19 | Physical mechanism underlying low-frequency *C*-*V* characteristics of dual Schottky junctions. a, The simplified *C*-*V* curves (bottom area) and the corresponding original *C*-*V* curves (top area) of dual Schottky junctions. The simplification is that the capacitance of junction L (C_1) and R (C_2) satisfies $C_1 = C_2 = 1$ F (independent on bias). The valley points in simplified curve and original curve locate exactly at the same position, and the valley point occurs only when $r = 1$. **b**, The capacitance variation of junction L ($C_1(V)$, dashed line) and R ($C_2(V)$, chain line) when the whole device based on dual Schottky junctions is biased. The solid line represents the corresponding original *C*-*V* curves. The peak occurs when the corresponding reverse-biased junction takes over the dominant role of *r* as the bias increases.

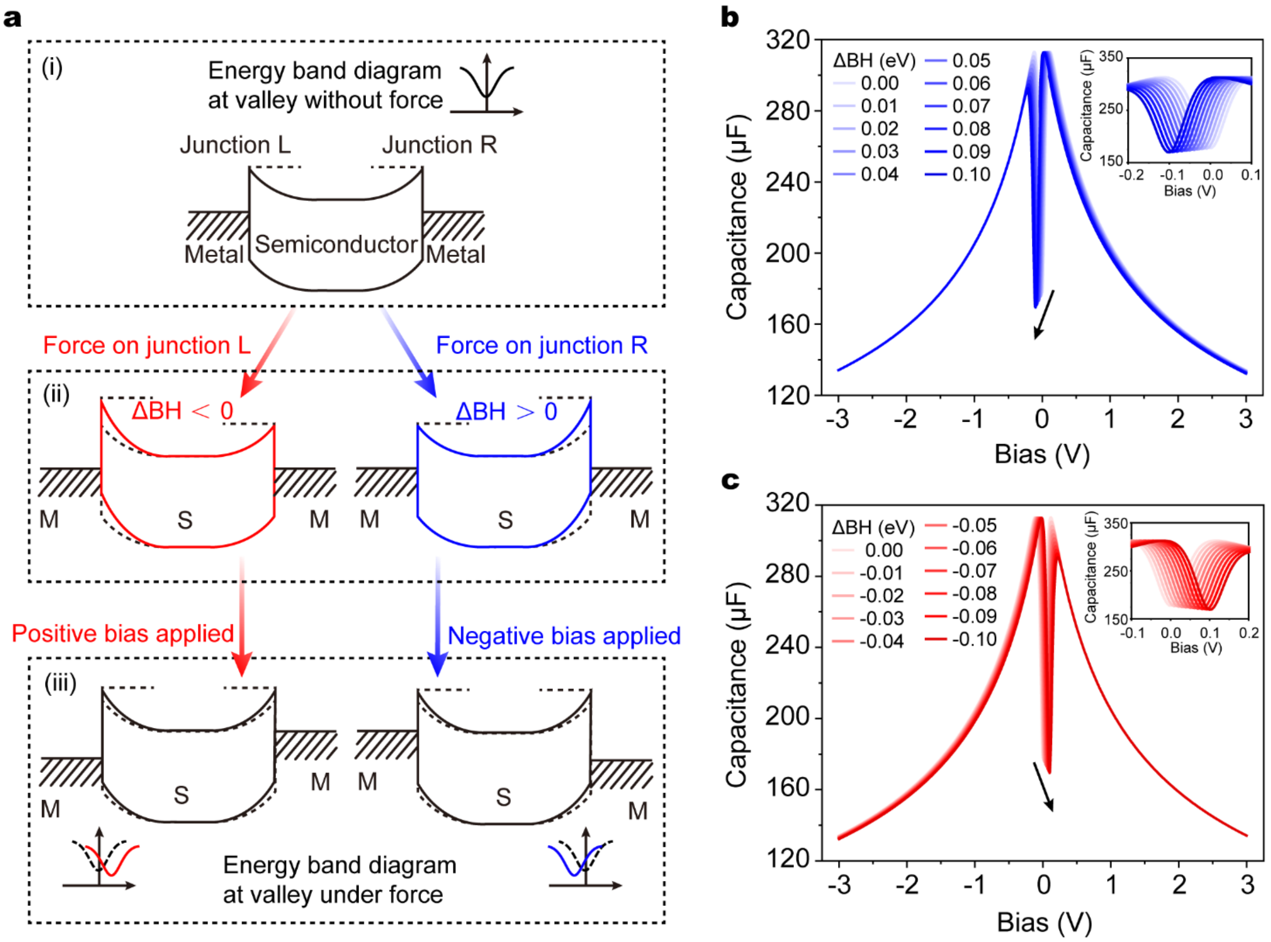

Supplementary Figure 20 | Modulation mechanism on valley of low-frequency *C-V* characteristics. a, Energy band diagrams corresponding to valley in low-frequency curves. The configuration of dual Schottky junctions is symmetric at valley point in *C-V* curves (i). When external force is applied on junction L (red) or R (blue), the energy band structure becomes asymmetric (ii) and positive or negative bias is applied to make energy band structure symmetric again (iii). Therefore, the valley point of the curve under loading force shifts to this bias, with interface barrier heights and widths of both junctions being broaden. **b**, **c**, Simulated low-frequency *C-V* characteristics of dual Schottky junctions with asymmetric configurations. The asymmetry in dual Schottky junctions is set by the differences between interface barrier heights (ΔBH = $\varphi_2 - \varphi_1$, where φ_1 and φ_2 refers to the interface barrier height of junction L and R, respectively).

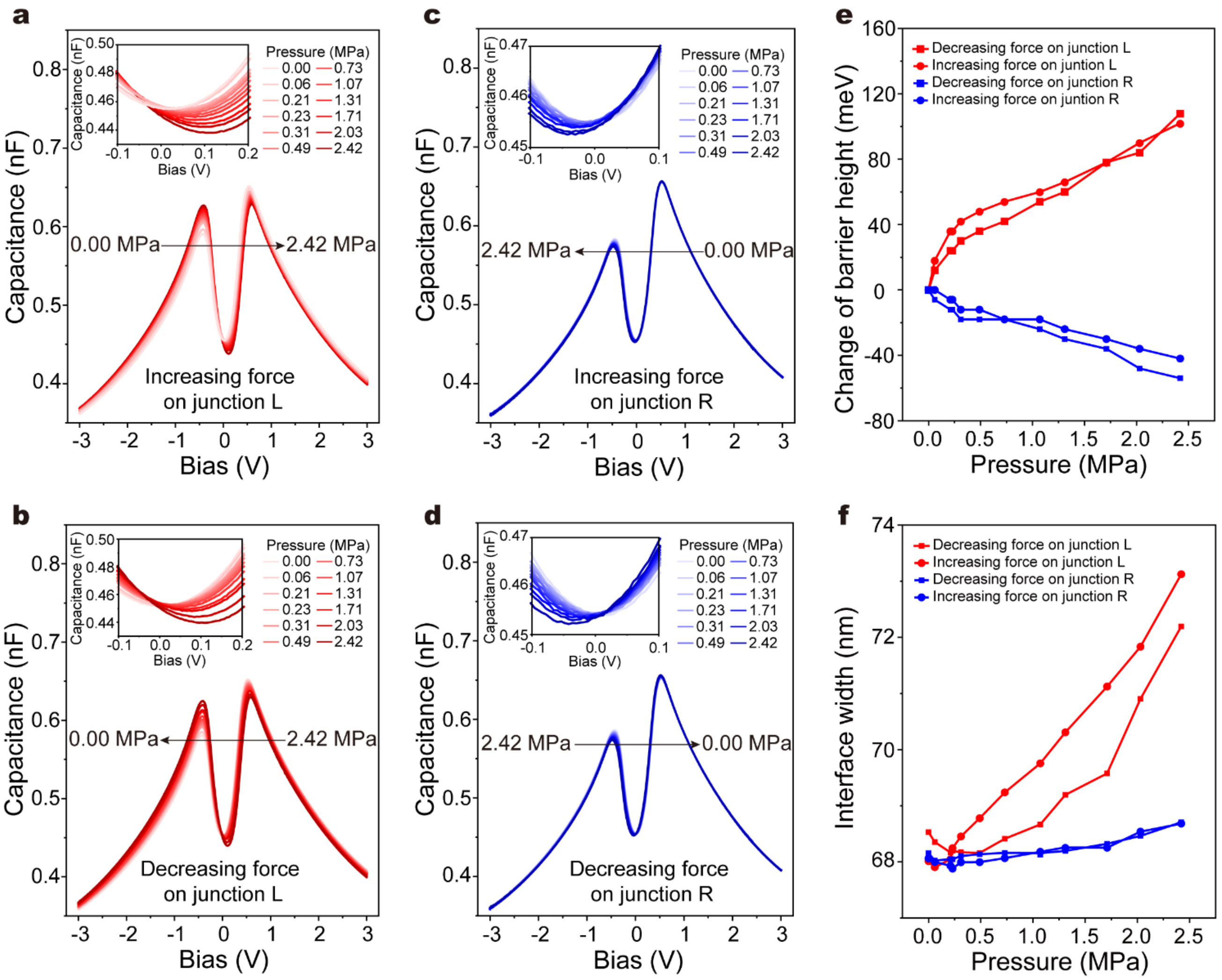

Supplementary Figure 21 | Calculations of interface barrier heights and widths of low-frequency *C*-*V* characteristics. a, **b**, The *C*-*V* characteristics with increasing (**a**) and decreasing (**b**) force applied to junction L. **c**, **d**, The *C*-*V* characteristics with increasing (**c**) and decreasing (**d**) force on junction R. **e**, Change of interface barrier heights in **a**-**d** under different loading forces. **f**, Interface widths in **a**-**d** under different loading forces.

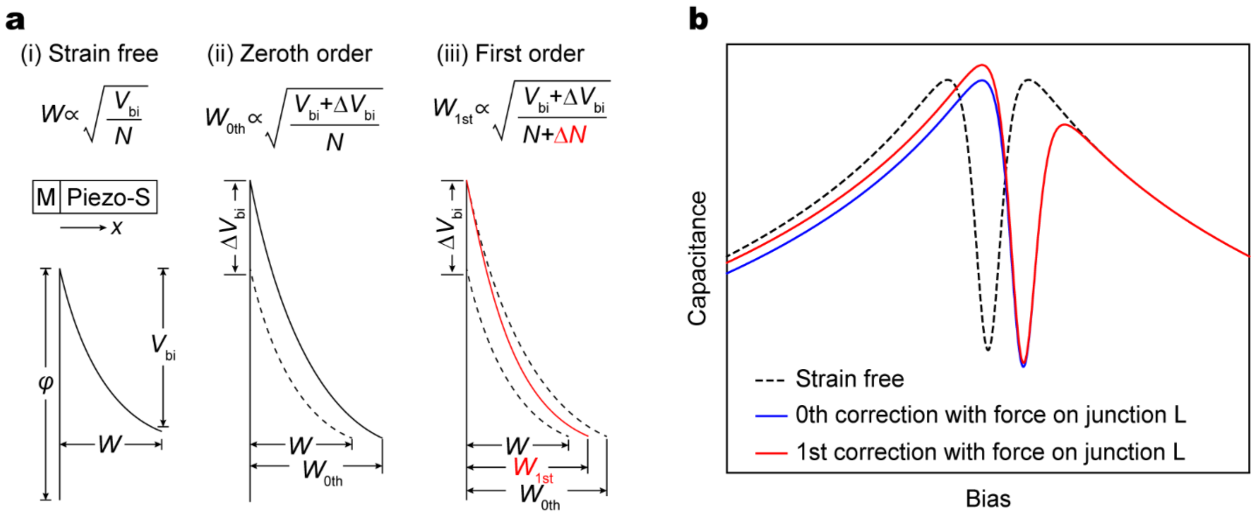

Supplementary Figure 22 | Correction on the theoretical model of dual Schottky junctions. a, Under strain free condition, the interface width (W) has a positive relation with built-in electric potential (V_{bi}) at zero bias (i). As the force increases, zeroth order correction, that the built-in electric potential is enhanced ($V_{\mathrm{bi}} + \Delta V_{\mathrm{bi}}$), is applied to represent the influence of polarization charges (ii). The first order correction, that the effective carrier concentration is also changed ($N + \Delta N$), is applied to further explain the increase of the peak value in low-frequency C-V curves (iii). The inset in **a**(i) shows the schematic of metal/piezo-semiconductor contact. **b**, The simulated low-frequency C-V curves of dual Schottky junctions under situations of strain free (dashed line), zeroth order correction (blue line) and first order correction (red line).

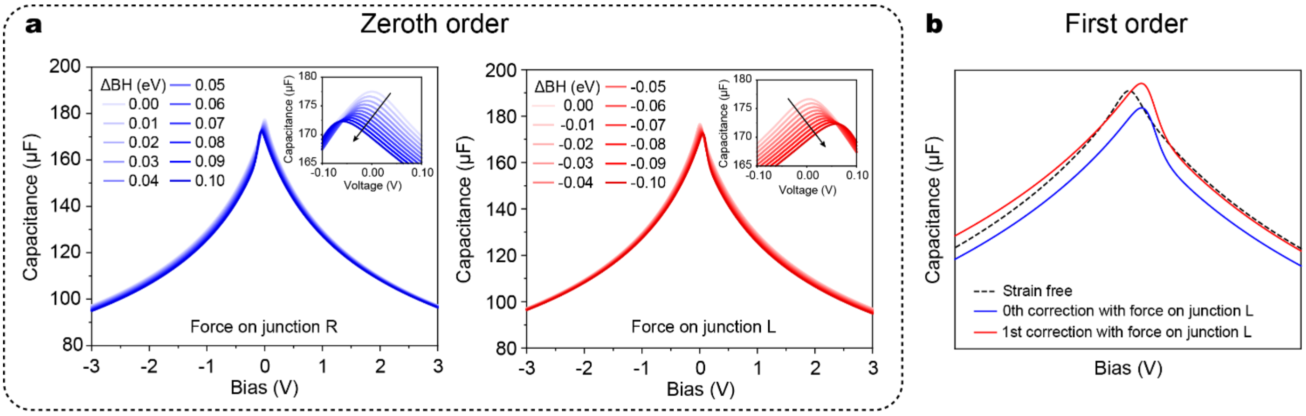

Supplementary Figure 23 | Theoretical simulation and the correction on the high-frequency *C-V* characteristics of dual Schottky junctions. a, **b**, Simulated high-frequency *C-V* characteristics of dual Schottky junctions with force applied to junction L (red) and R (blue). The force-induced change is introduced by changing $\Delta BH = \varphi_2 - \varphi_1$, where φ_1 and φ_2 refers to the interface barrier height of junction L and R, respectively. **c**, The simulated low-frequency *C-V* curves of dual Schottky junctions under situations of strain free (dashed line), zeroth order correction (blue line) and first order correction (red line).

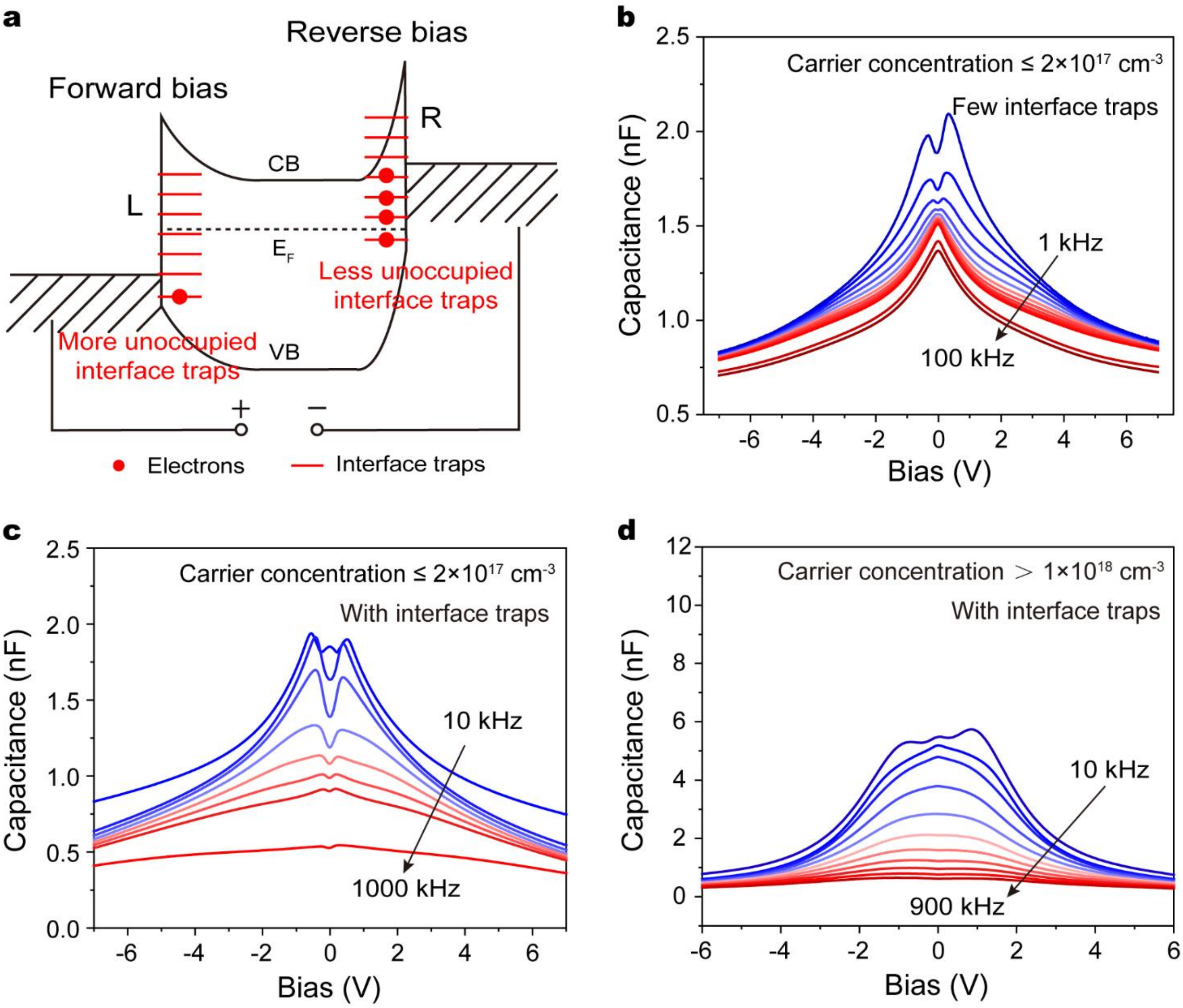

Supplementary Figure 24 | *C-V* characteristics of GaN with interface traps. a, Interface traps in dual Schottky junctions L and R. The interface of forward-biased junction L possesses more unoccupied traps than the reverse-biased junction R. **b-d**, The dispersive *C-V* characteristics of dual Schottky junctions based on GaN with low carrier concentration and few interface traps (**b**), low carrier concentration and interface traps (**c**), and high carrier concentration and interface traps (**d**).

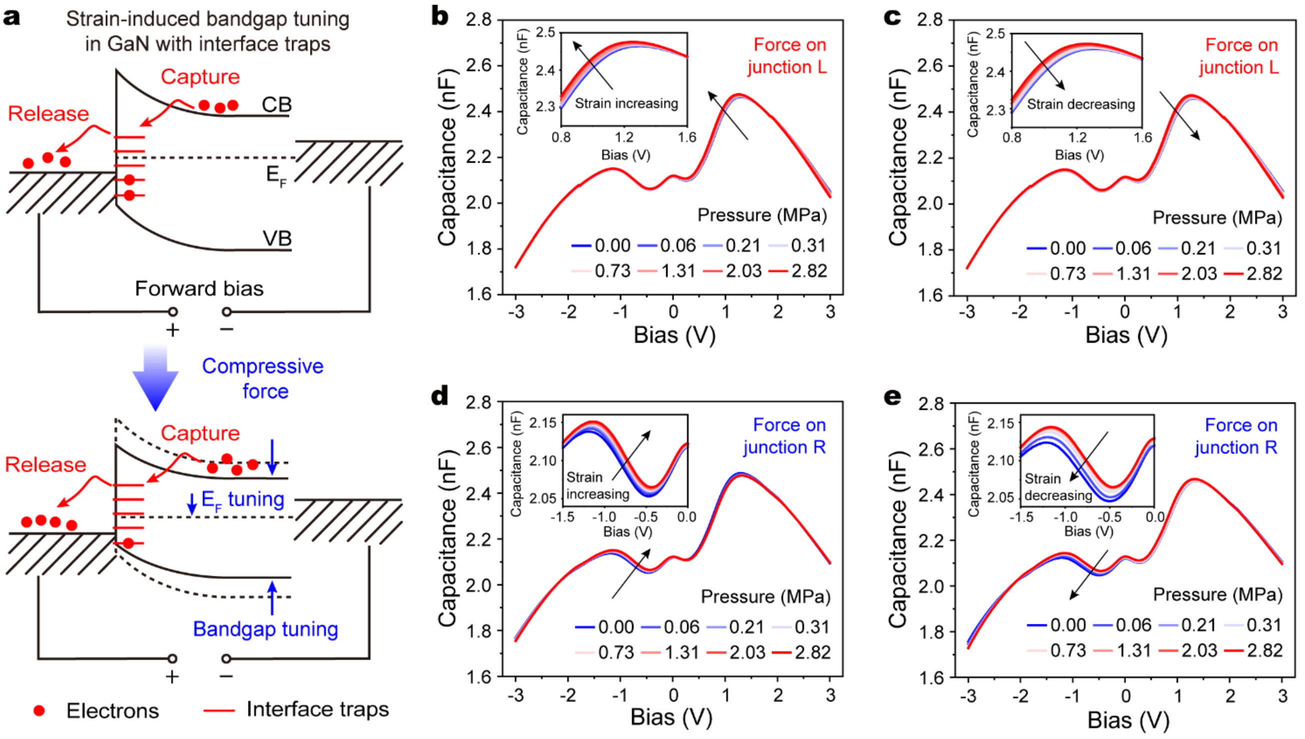

Supplementary Figure 25 | Strain induced bandgap tuning in GaN with interface traps. a, Carriers capture and release at interface traps under strain-induced bandgap tuning effect. With compressive force applied along with polarization *c*-axis of piezoelectric *n*-GaN, the conduction band significantly decreases while the valence band only slightly increases, leading to the reduction in Fermi level. More unoccupied traps are generated. **b-e**, Change of excess capacitance with increasing loading force applied on junction L (**b**) and R (**d**), and with decreasing loading force applied on junction L (**c**) and R (**e**). The GaN used in measurements is of high carrier concentration and interface traps.

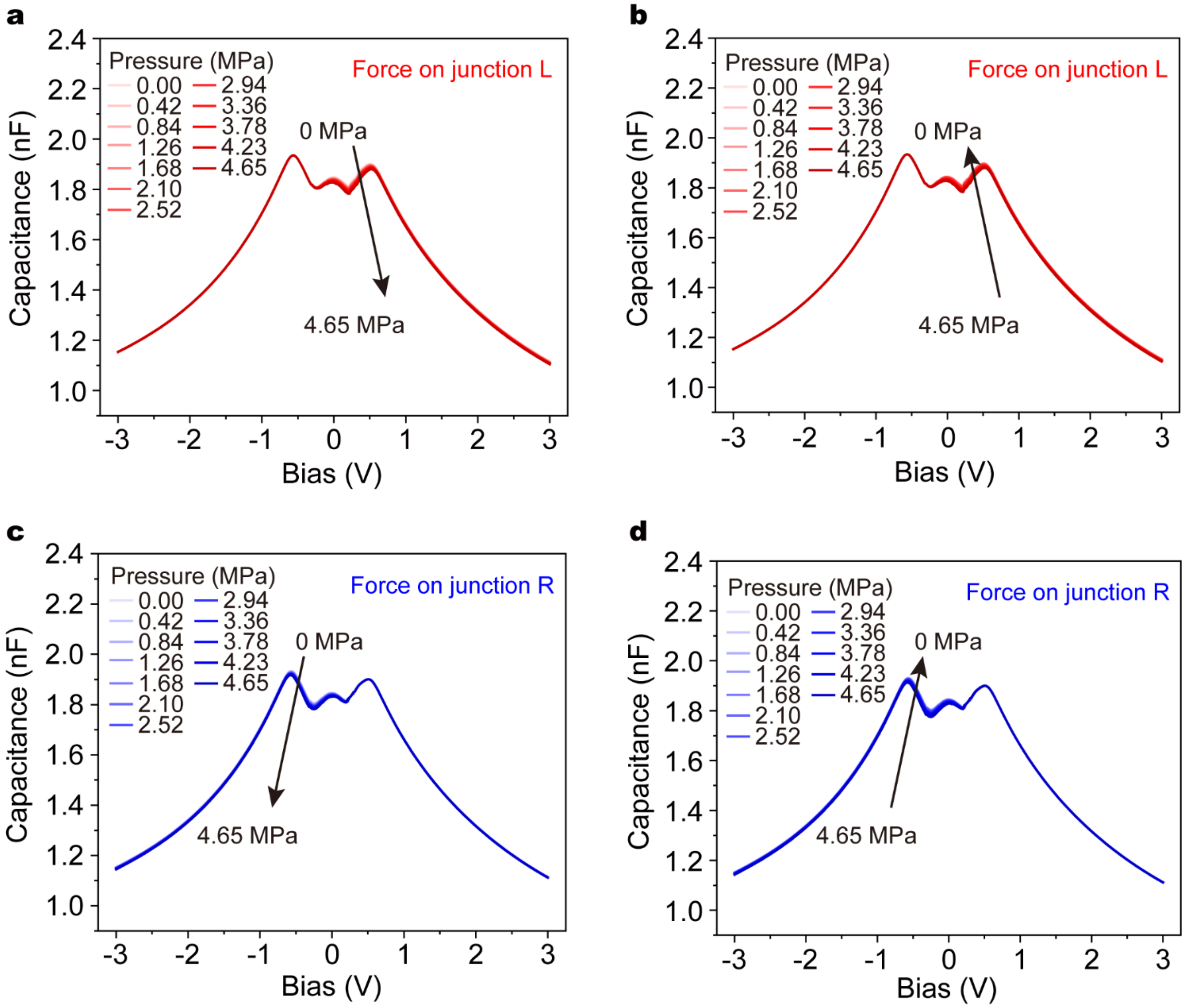

Supplementary Figure 26 | Capacitive piezotronic effect in GaN with interface traps. a, **b**, Variation in excess capacitance under force applied to junction L. Measurements were conducted with increasing loading force (**a**) and decreasing loading force (**b**). **c**, **d**, Variation in excess capacitance under force applied to junction R. Measurements were conducted with increasing loading force (**c**) and decreasing loading force (**d**). The excess capacitance shows opposite variation under loading force compared to results in Supplementary Fig. 25, resulting from the completely different influence on Schottky interface between capacitive piezotronic effect and strain-induced bandgap tuning effect. The GaN used in measurements is of low carrier concentration and interface traps.

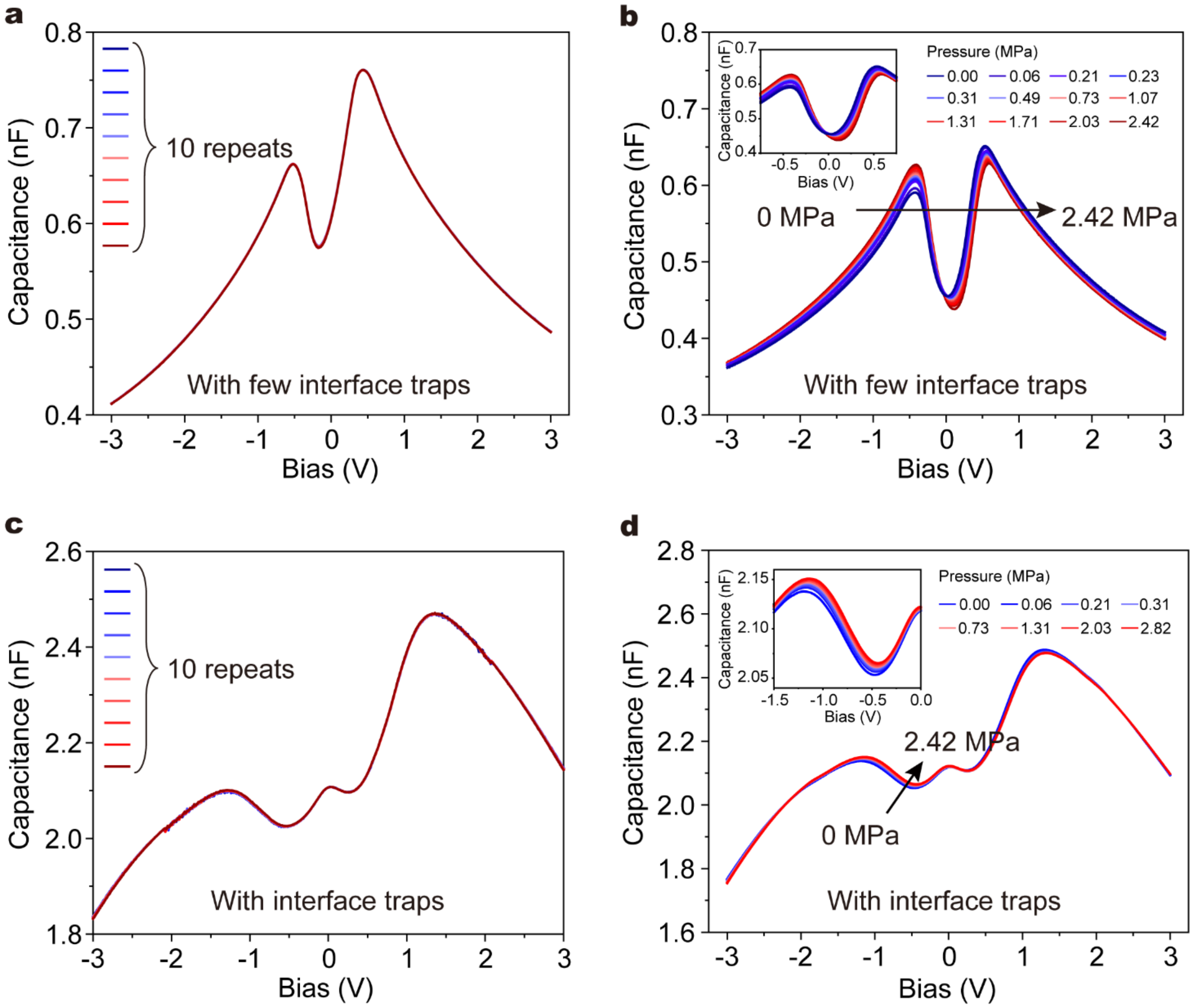

Supplementary Figure 27 | Repetitive measurements of *C-V* characteristics of dual Schottky junctions. **a**, **b**, The repetitive *C-V* measurements sweeping from –3 V to 3 V in GaN with few interface traps. The situation without force applied (**a**) shows almost no change compared to that under loading force (**b**). **c**, **d**, The repetitive *C-V* measurements sweeping from –3 V to 3 V in GaN with interface traps. The situation without force applied (**c**) shows almost no change compared to that under loading force (**d**). The results demonstrate that in our experiments the strain-induced effects, primarily capacitive piezotronic effect and stain-induced bandgap tunning, are the main factors to modulate the *C-V* characteristics rather than other electrical factors introduced by repetitive measurements.

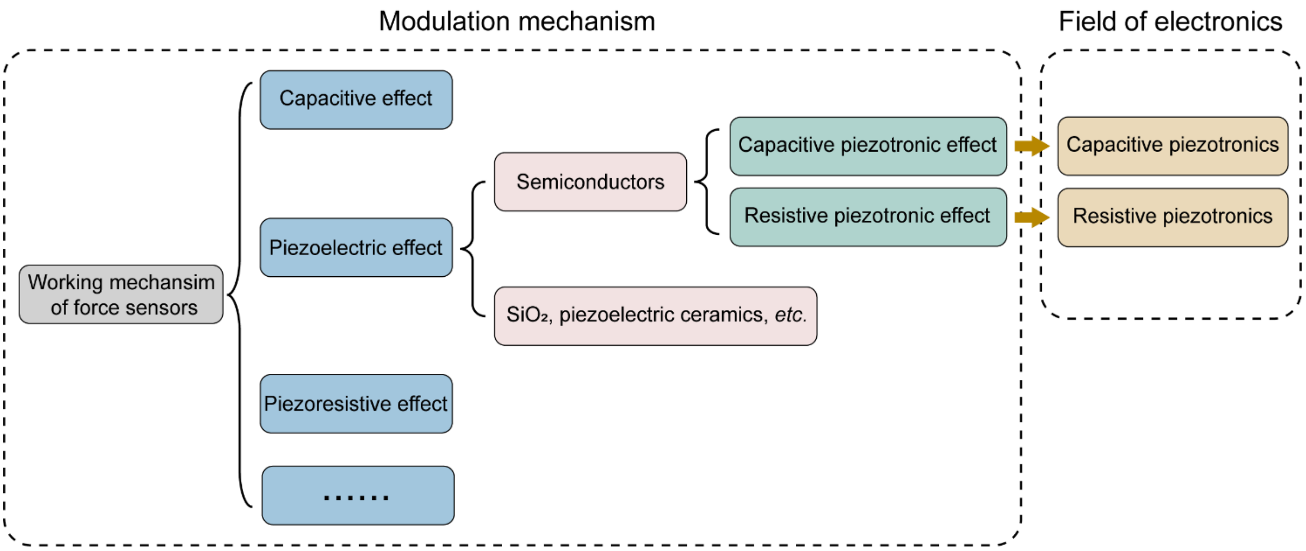

Supplementary Figure 28 | Working mechanism of force sensors. Working mechanisms of force sensors generally include capacitive effect, piezoelectric effect, piezoresistive effect and so on. Among these, piezoelectric effect can be divided into two parts according to the materials of sensors, traditional piezoelectric effect based on conventional materials such as SiO_2, piezoelectric ceramics, etc. and piezotronic effect coupling piezoelectricity and semiconductor properties. Previous work on resistive piezotronic effect focus on the change in resistance of semiconductor device while capacitive piezotronic effect proposed in our work focus on the change in capacitance. Moreover, utilizing resistive/capacitive piezotronic effect to control capacitance of devices, so as to achieve diverse functions in DC/AC systems is defined as resistive/capacitive piezotronics, which refers to a distinctive field of electronics.

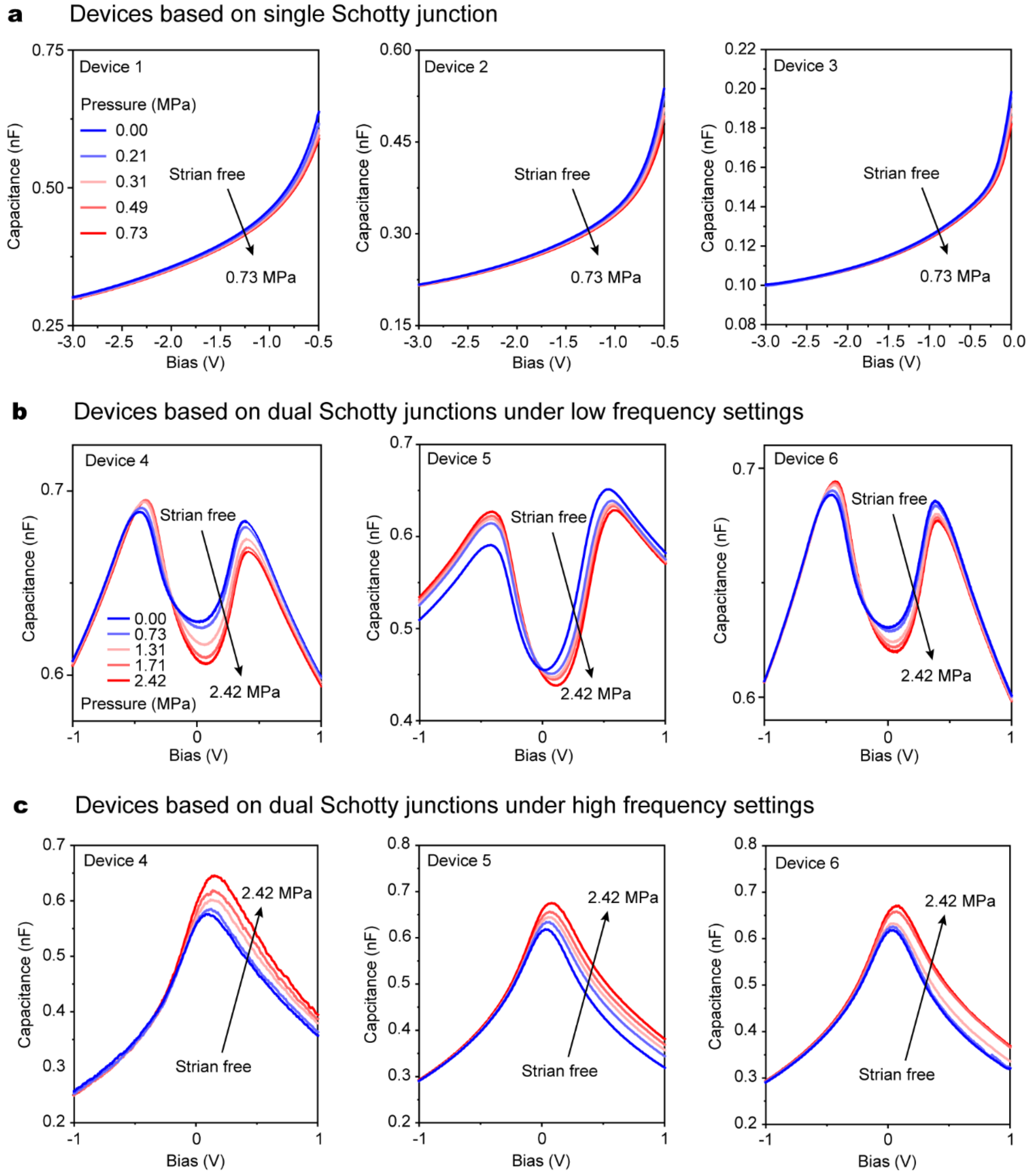

Supplementary Figure 29 | Force-dependent *C-V* characteristics of multiple independent devices. **a**, Force-dependent *C-V* characteristics of devices 1-3 based on single Schottky junctions. **b**, Force-dependent low-frequency (1 kHz) *C-V* characteristics of devices 4-6 based on dual Schottky junctions. **c**, Force-dependent high-frequency (10 kHz) *C-V* characteristics of devices 4-6 based on dual Schottky junctions.

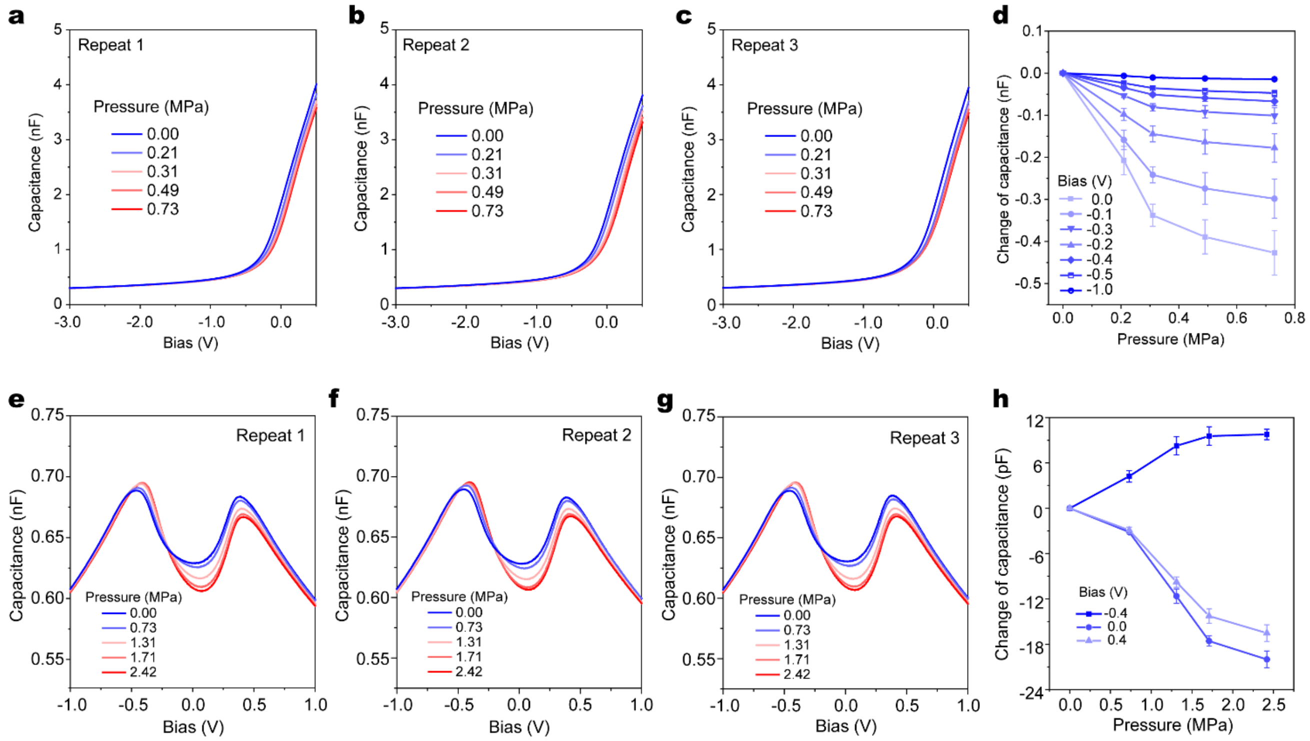

Supplementary Figure 30 | Repeat *C*-*V* measurements. a-c, Repeat *C*-*V* measurements on single Schottky junction based device. **d,** The force-induced change in capacitance of single Schottky junction based device under different bias. **e-f**, Repeat *C*-*V* measurements on dual Schottky junctions based device. **d,** The force-induced change in capacitance of dual Schottky junctions based device under different bias.

References

1 Murata. SCB10H series pressure sensor elements. https://www.murata.com/en-us/products/sensor/pressure/pressure-elements (2013).

2 Protron Mikrotechnik. Capacitive pressure sensors. https://www.protron.de/products/products_sensors_e.htm (2014).

3 Korkut, H. Semiconductor type dependent comparison of electrical characteristics of Pt/InP structures fabricated by magnetron sputtering technique in the range of 20-400 K. *Nano-Micro Lett.* **5**, 34-39 (2013).

4 Anderson, W. A. & Milano, R. A. *I-V* characteristics for silicon Schottky solar cells. *Proc. IEEE* **63**, 206-208 (1975).

5 Cohen, L. D. Microwave characterization of the properties and performance of GaAs Schottky barrier mixer diodes. *Proc. IEEE* **59**, 288-289 (1971).

6 Van Opdorp, C. & Kanerva, H. K. J. Current-voltage characteristics and capacitance of isotype heterojunctions. *Solid-State Electron.* **10**, 401-421 (1967).

7 Sze, S. M., Coleman, D. J. & Loya, A. Current transport in metal-semiconductor-metal (MSM) structures. *Solid-State Electron.* **14**, 1209-1218 (1971).

8 Sze, S. M., Li, Y. & Ng, K. K. *Physics of Semiconductor Devices*. (John Wiley & Sons, 2021).

9 Ho, P. S., Yang, E. S., Evans, H. L. & Wu, X. Electronic states at silicide-silicon interfaces. *Phys. Rev. Lett.* **56**, 177-180 (1986).

10 Vasudev, P. K., Mattes, B. L., Pietras, E. & Bube, R. H. Excess capacitance and non-ideal Schottky barriers on GaAs. *Solid-State Electron.* **19**, 557-559 (1976).

11 Werner, J., Levi, A. F. J., Tung, R. T., Anzlowar, M. & Pinto, M. Origin of the excess capacitance at intimate Schottky contacts. *Phys. Rev. Lett.* **60**, 53-56 (1988).

12 Chattopadhyay, P. & Raychaudhuri B. Frequency dependence of forward capacitance-voltage characteristics of Schottky barrier diodes. *Solid-State Electron.* **36**, 605-610 (1993).

13 Chuang, S. L. Optical gain of strained wurtzite GaN quantum-well lasers. *IEEE J. Quantum Electron.* **32**, 1791-1800 (1996).

14 Chuang, S. L. & Chang, C. S. k·p method for strained wurtzite semiconductors. *Phys. Rev. B* **54**, 2491-2504 (1996).

15 Nardelli, M. B., Rapcewicz, K. & Bernholc, J. Strain effects on the interface properties of nitride semiconductors. *Phys. Rev. B* **55**, R7323-R7326 (1997).

16 Cho, Y. & Mandi, Y. Dynamic measurement of capacitance variation of piezoelectric ceramics with stress. *Jpn. J. Appl. Phys.* **34**, 1591(1995).
